# Hydrazone-Linked Covalent Organic Framework Catalyst via Efficient Pd Recovery from Wastewater

**DOI:** 10.1021/acsami.4c07706

**Published:** 2024-08-21

**Authors:** Mahira Bashri, Sushil Kumar, Pallab Bhandari, Sasi Stephen, Matthew J. O’Connor, Safa Gaber, Tina Škorjanc, Matjaž Finšgar, Gisha Elizabeth Luckachan, Blaž Belec, Emad Alhseinat, Partha Sarathi Mukherjee, Dinesh Shetty

**Affiliations:** † Department of Chemistry, Khalifa University of Science & Technology, Post Office Box 127788, Abu Dhabi, United Arab Emirates; ‡ Department of Inorganic and Physical Chemistry, Indian Institute of Science, Bangalore 560012, India; § New York University Abu Dhabi, Post Office Box 129188, Abu Dhabi, United Arab Emirates; ∥ Materials Research Laboratory, University of Nova Gorica, Vipavska 11c, 5270 Ajdovscina, Slovenia; ⊥ University of Maribor, Smetanova ulica 17, 2000 Maribor, Slovenia; # Department of Chemical and Petroleum Engineering, Khalifa University of Science & Technology, Post Office Box 127788, Abu Dhabi, United Arab Emirates; ∇ Center for Catalysis & Separations (CeCaS), Khalifa University of Science & Technology, Post Office Box 127788, Abu Dhabi, United Arab Emirates

**Keywords:** covalent organic frameworks, sustainability, catalysis, palladium adsorption, water purification

## Abstract

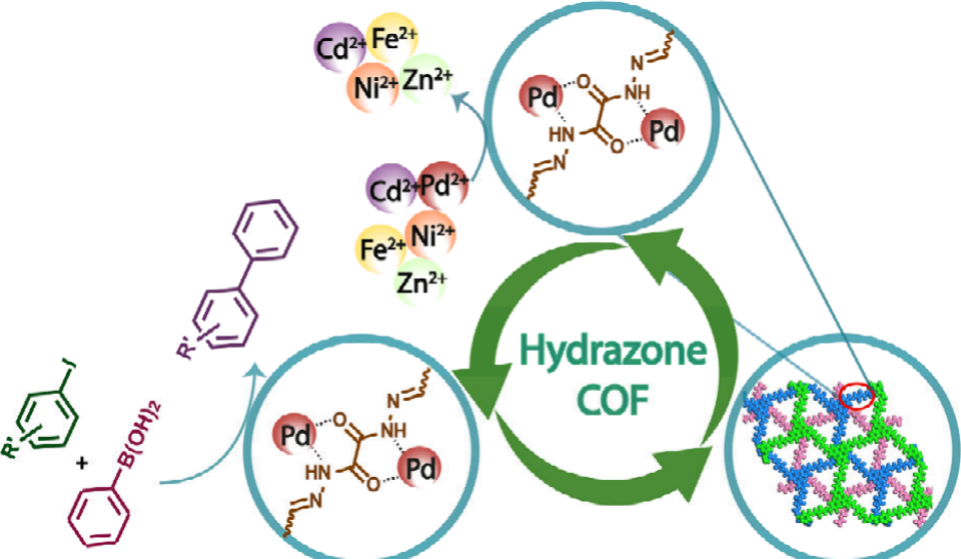

Global consumption and discharge of palladium (Pd) have raised environmental concerns but also present an opportunity for the sustainable recovery and reuse of this precious metal. Adsorption has proven to be an efficient method for the selective recovery of Pd from industrial wastewater. This study investigated a hydrazone-linked covalent organic framework (**Tfpa-Od** COF) as a potential material for the high-affinity adsorption of Pd^2+^ ions from wastewater, achieving a *K*_d_ value of 3.62 × 10^6^ mL g^–1^. The electron-rich backbone of the COF contributes to its excellent selective removal efficiency (up to 100%) and adsorption capacity of 372.59 mg g^–1^. Furthermore, the Pd-adsorbed COF was evaluated as a sustainable catalyst for the Suzuki–Miyaura coupling reaction, demonstrating good catalytic conversion and recyclability. This work attempts to showcase a protocol for reusing waste palladium generated in water to fabricate heterogeneous catalysts and, thereby, promote the circular economy concept.

## Introduction

Palladium (Pd) is a highly sought-after metal due to its unique physicochemical properties and extensive industrial applications.^1,2^ It plays a crucial role in reducing automotive emissions, including hydrocarbons, carbon monoxide, and nitrous oxide, through its use in Pd-based catalytic converters, which help mitigate the release of these toxic gases into the environment.^3^ However, the rapid and extensive industrial consumption of Pd has led to a significant increase in the presence of Pd (in the form of deactivated catalysts and electronic waste) in water resources.^4^ Although the concentration of Pd in the environment, particularly in aquatic ecosystems, is currently lower than toxic levels, its accumulation over time can disrupt flora, fauna, and eventually human health.^5,6^ Conversely, conventional extraction of Pd from natural ores is becoming insufficient to meet global demand due to the declining availability of processable forms.^7,8^ Consequently, the recovery and reuse of Pd from secondary sources, such as industrial wastewater, can achieve two critical objectives: promoting sustainability and aiding in water remediation.^9^ However, the selective removal of Pd from industrial wastewater is challenging due to the presence of high concentrations of competitive interfering metal ions and the overall complexity of polluted water. Although various physicochemical techniques, such as solvent extraction, membrane sieving, coagulation/precipitation, ion exchange, and reverse osmosis, have been employed for Pd recovery from wastewater, these methods often suffer from limitations regarding performance, cost efficiency, specificity, and the generation of toxic waste byproducts.^10−14^ Notably, the adsorption technique has shown promising potential for the selective and efficient recovery of precious metals. Conventional sorbents, such as carbon-based materials, silica, and metal sulfides, show less efficiency in adsorption capacity, kinetics, and affinity parameters. The notable discrepancies in the performance metrics of such materials are due to their less scope in functional tunability.^15−19^ Meanwhile, porous amorphous polymers are employed for the adsorptive removal of Pd in wastewater through either coordinative (ligand exchange) or electrostatic interactions with functionalities, such as thiourea,^20^ pyrazole,^21^ pyridyl, and amino^22^ units, present in the polymer backbone. While these systems are rich in functional sites to anchor Pd, they often suffer from inefficient exposure of such active sites due to their inherent amorphous nature and less ordered porosity. The chelation chemistry and porous nature of materials are key factors in controlling the adsorption efficiency and kinetics.

With the advent of reticular chemistry, which allows for structural and property tunability and the incorporation of diverse coordination sites within the pore interiors, covalent organic frameworks (COFs) have demonstrated exceptionally high adsorption capacities for many toxic metals in water.^23−25^ For Pd recovery, COFs leverage electrostatic or coordinative interactions, achieved either through synthesizing ionic COFs with an electron-rich backbone or post-synthetic grafting of ionic side-chain functionalities.^26−28^ However, existing COFs often lack efficient uptake of Pd ions, particularly regarding their adsorption capacity and kinetics. Thus, exploring novel coordination environments within COF structures for Pd recovery is a promising research direction. Additionally, Pd-embedded (metal-doped) COFs are utilized for efficient heterogeneous catalysis.^29,30^ Combining these approaches, using COFs as sorbents for Pd recovery from wastewater followed by their application in catalysis, can significantly contribute to sustainable catalyst development and support the circular economy.

Considering the coordination ability of Pd^2+^ ions toward electron-rich species, we selected 1,3,5-tris(4-formylphenyl)amine (Tfpa) and “flexible alkylamine” oxalyl dihydrazide (Od) as precursors to generate a hydrazone-linked crystalline **Tfpa-Od** COF, which exhibits selective metal ion uptake from wastewater. The resulting material demonstrated rapid uptake kinetics with 100% efficiency and an outstanding adsorption capacity (*q*_max_ = 372.59 mg g^–1^) among COF-based adsorbent materials for Pd recovery.^26,28,31^ The Pd-adsorbed COF (**Pd@Tfpa-Od**) was further utilized as a sustainable heterogeneous organocatalyst for the synthesis of biaryls via the Suzuki–Miyaura coupling reaction, achieving excellent catalytic conversion. This study highlights the synergistic potential of COFs in both precious metal ion recovery from wastewater and their fabrication as efficient heterogeneous catalysts.

## Experimental Section

### Synthesis of **Tfpa-Od** COF

The monomers Od (0.2732 mmol) and Tfpa (0.1822 mmol) were placed in a 100 mL pressure vial and dissolved using a mesitylene and 1,4-dioxane mixture (1:4, v/v). Subsequently, 1 mL of 6 M acetic acid was added to the solution, and the vial was sealed and heated at 120 °C for 72 h. The resulting yellow precipitate was collected and thoroughly washed with deionized water, tetrahydrofuran (THF), and acetone. The product was then dried overnight in an oven at 90 °C, giving the final material with a yield of 94%.

### Pd^2+^ Adsorption Experiments

#### Adsorption Kinetics

A total of 5 mg of **Tfpa-Od** COF was introduced to 200 mL of a 10 ppm of Pd^2+^ solution. Samples of 1 mL each were collected between 0 and 1 h at various time points. These samples were filtered using 0.45 μm Nylon syringe filters. The filtrate solutions were then analyzed using inductively coupled plasma mass spectrometry (ICP–MS) to determine the residual metal concentration. The results were fitted to a pseudo-second-order kinetic model (eq 1 in section 4 of the Supporting Information).

#### Adsorption Isotherm

To obtain the isotherm for adsorption, 10 mL of Pd^2+^ solutions with varying concentrations ranging from 2 to 500 ppm were prepared. Then, 5 mg of **Tfpa-Od** COF was added to each solution, and the mixture was stirred until equilibrium was reached. The changes in Pd^2+^ concentrations were monitored via ICP–MS analysis. The obtained results were fitted to the Langmuir adsorption isotherm model (eq 5 in section 4 of the Supporting Information)

#### Selectivity Test

To obtain selective Pd^2+^ adsorption, a metal ion solution containing 10 ppm each of Pd^2+^, Fe^2+^, Ni^2+^, Zn^2+^, and Cd^2+^ was prepared. A total of 5 mg of the **Tfpa-Od** COF adsorbent was added and stirred until equilibrium was reached. The samples obtained were filtered using 0.45 μm Nylon syringe filters before the ICP–MS analysis.

#### Regeneration Experiments

The **Tfpa-Od** COF regeneration experiments were carried out by adsorption using 10 ppm of Pd^2+^ solutions. The desorption experiment was done by overnight stirring **Pd**^**2+**^**@Tfpa-Od** with stripping solution (0.2 M thiourea and 0.2 M HCl at pH 2). Yellow **Tfpa-Od** was filtered, washed several times in distilled water, and reused for further adsorption cycles. The experiment was repeated for up to 5 cycles. The samples were analyzed via ICP–MS, and adsorption capacities were calculated.

#### Effect of pH

The effect of pH in the adsorption of Pd over **Tfpa-Od** (5 mg) was investigated by preparing 10 ppm of Pd^2+^ solutions at varying pH (2, 4, 6, 7, and 8) and treated overnight. The adsorption was monitored by analyzing the filtrates via ICP–MS analysis, and uptake efficiencies were calculated.

#### Reduction of **Pd**^**2+**^**@Tfpa-Od** into **Pd-NP@Tfpa-Od**

A 0.5 M NaBH_4_ solution was added dropwise carefully into a MeOH/H_2_O (1:1, v/v) solution of **Pd**^**2+**^**@Tfpa-Od**. The reaction was left to stir at 200 rpm for 24 h under an argon atmosphere. The solid material was centrifuged and washed with ethanol several times. The product was dried in an oven at 80 °C for 12 h to obtain a greenish yellow powder of **Pd-NP@Tfpa-Od**, which was further utilized as a catalyst.

#### General Procedure for Catalysis

In a 50 mL round-bottom (RB) flask, aryl iodide (0.5 mmol), phenyl boronic acid (91.5 mg, 0.75 mmol), Cs_2_CO_3_ (325.8 mg, 1 mmol), and 2 mg of **Pd-NP@Tfpa-Od** catalyst were taken in 7 mL of 1,4-dioxane. The resulting mixture was degassed under an inert atmosphere and stirred for 24 h at 90 °C. After completion of the reaction, the solid catalyst was removed by filtration. The catalyst was washed with 1,4-dioxane/water and dried in vacuum for the next cycle of use. The 1,4-dioxane solvent was completely evaporated, and the residue was extracted with CHCl_3_ from the mixture of CHCl_3_/water solvents. The organic part was washed with water multiple times and dried over anhydrous Na_2_SO_4_, followed by complete solvent removal. ^1^H nuclear magnetic resonance (NMR) analyzed the resulting crude mass to calculate the product conversion. Moreover, the crude product was purified by column chromatography (silica gel, 100 mesh) in hexane/chloroform to obtain an isolated yield. Further details are available in section 5 of the Supporting Information.

## Results and Discussion

**Figure 1:**
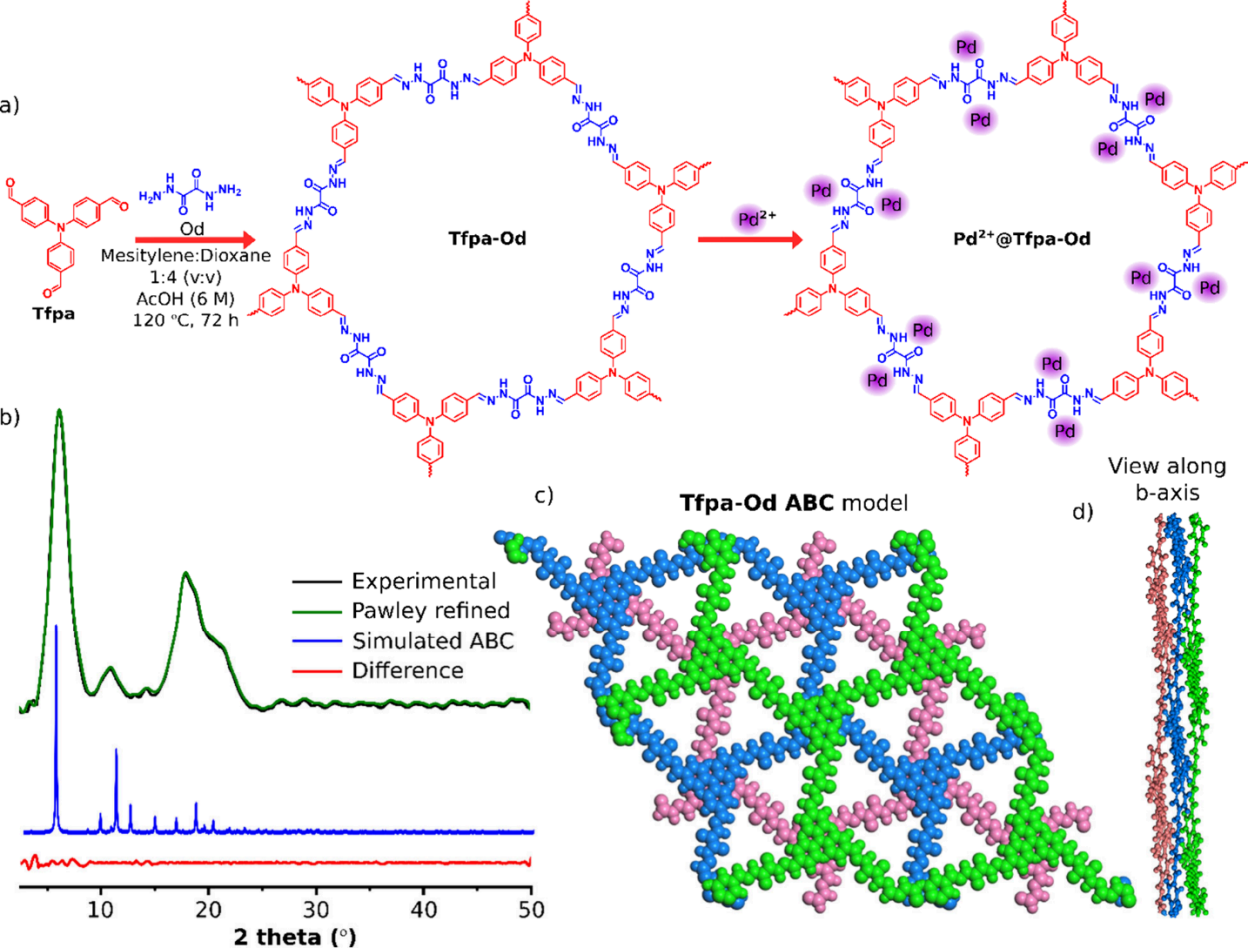
(a) Schematic representation for the synthesis of **Tfpa-Od** and **Pd**^**2+**^**@Tfpa-Od**, (b) comparison of the experimental PXRD pattern of **Tfpa-Od** with simulated ABC stacking, (c) ABC-simulated model view of **Tfpa-Od**, and (d) its view along the *b* axis showing the interplanar orientations.

**Tfpa-Od** COF was synthesized via the Schiff base condensation reaction between Tfpa and Od using the solvothermal method (Figure 1a). First, the pressure tube was charged with monomers (Tfpa and Od) dissolved in a mixture of mesitylene and 1,4-dioxane and allowed to condense under the catalytic action of acetic acid at 120 °C for 72 h to obtain **Tfpa-Od**. As-synthesized **Tfpa-Od** was isolated as a bright yellow powder, purified by thorough washing with water, THF, and acetone, and dried under a vacuum before detailed characterizations. The formation of **Tfpa-Od** was first confirmed by Fourier transform infrared (FTIR) spectroscopic studies (Figure S3 of the Supporting Information), where the appearance of a peak at 1591 cm^–1^ corresponds to −C=N vibration bands, confirming the successful formation of imine bonds. Moreover, in comparison to the spectra of **Tfpa-Od**, the intensity of the −C=O stretching band at 1668 cm^–1^ for Tfpa was reduced, whereas the −N–H stretching band in the range of 3200–3300 cm^–1^ for Od disappeared, indicating the consumption of monomers.^32,33^

The **Tfpa-Od** crystallinity was determined using powder X-ray diffraction (PXRD) studies (Figure 1b). The sharpness of the peaks in the PXRD profile indicates the crystalline nature of the material, with the low angle peak at 2θ = 6.2°, which is assigned to the diffraction from 110 facets, whereas a broad peak present in the range of 2θ = 15–25° corresponds to the diffraction from 001 facets. On the basis of the PXRD pattern and monomer structures, the molecular structure of **Tfpa-Od** was simulated for AA-eclipsed, AB, and ABC-staggered conformation models using Material Studio (see Figures S1 and S2 of the Supporting Information). The experimental PXRD pattern was compared to all three simulated conformation models and found to be best correlated with the simulated ABC-stacking model (panels b, c, and d of Figure 1). Following the Pawley refinement, the unit cell parameters for the ABC model, space group *P*1, were calculated as *a* = 32.29 Å, *b* = 30.73 Å, *c* = 9.88 Å, α = 86.84°, β = 88.22°, and γ = 115.25°, respectively.

**Figure 2:**
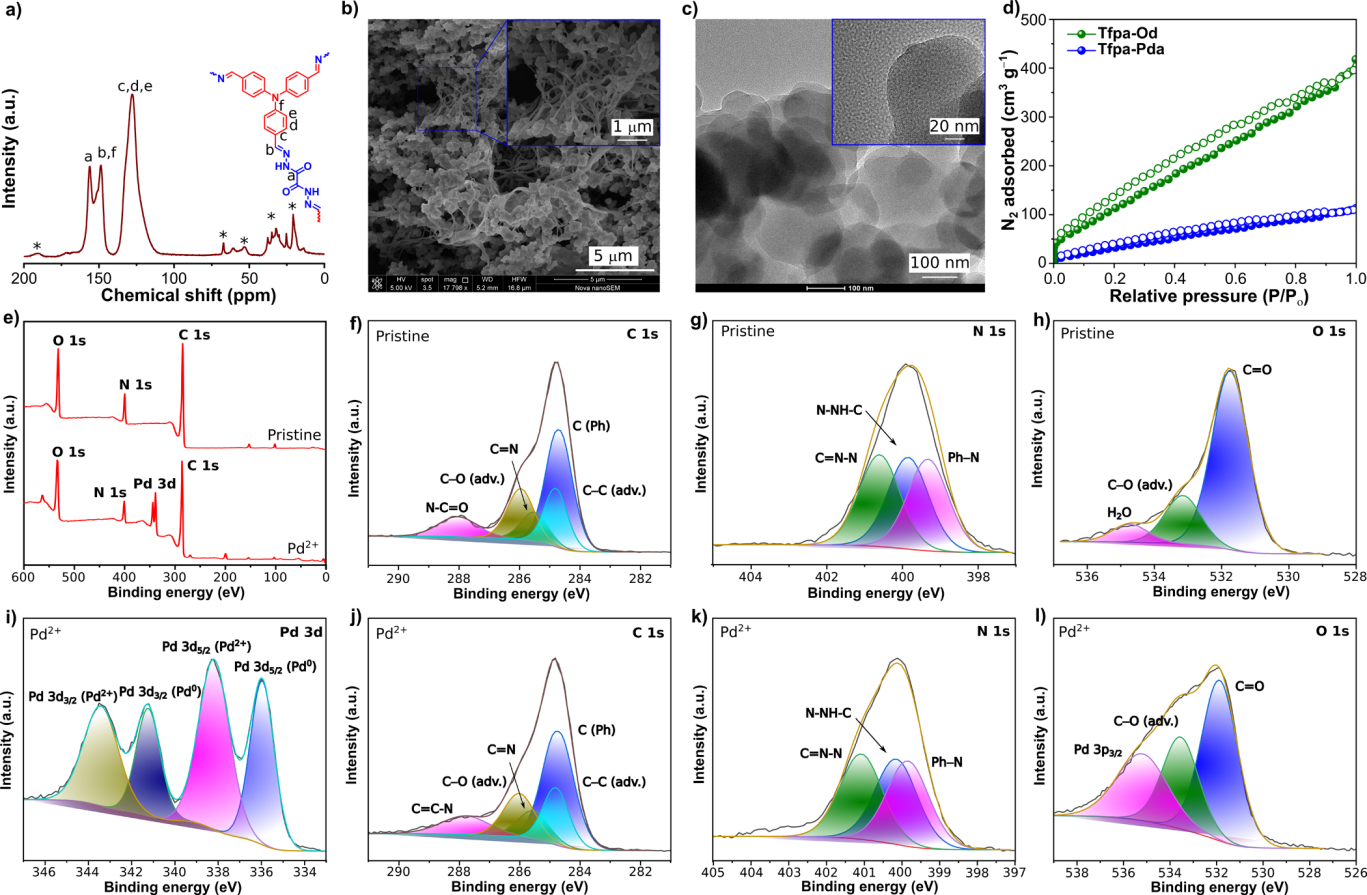
(a) ^13^C CP/MAS NMR spectrum of **Tfpa-Od**, (b and c) SEM and TEM images of **Tfpa-Od**, (d) nitrogen gas adsorption isotherm for **Tfpa-Od** in comparison to imine-linked **Tfpa-Pda**, (e) XPS survey spectra for **Tfpa-Od** before and after Pd uptake, (f–h) deconvoluted XPS spectra of **Tfpa-Od** for C 1s, N 1s, and O 1s, and (i–l) deconvoluted XPS spectra of **Pd**^**2+**^**@Tfpa-Od** for C 1s, N 1s, and O 1s, respectively.

Furthermore, molecular level information was obtained from solid-state ^13^C cross-polarization (CP) magic angle spinning (MAS) NMR analysis shown in Figure 2a. The spectrum exhibits a strong resonance peak at 128 ppm corresponding to aromatic carbon (C=C) of benzene rings, whereas the peak at 157 ppm corresponds to −C=O present in the **Tfpa-Od** skeleton. Notably, the peak observed at 149 ppm confirms the formation of −C=N– bonding in **Tfpa-Od**.^32,33^ The thermal stability of the material was investigated via thermogravimetric analysis (TGA) in an inert N_2_ atmosphere, which indicates thermal stability up to ∼300 °C (Figure S13a of the Supporting Information).

The bulk morphology of **Tfpa-Od** was investigated using scanning electron microscopy (SEM) and transmission electron microscopy (TEM) studies. SEM images of **Tfpa-Od** showed uniform long rod-shaped structures (∼2.5 μm long and 150–200 nm thickness), terminating in small plate-like (∼500 nm wide) expansions, suggesting a dendritic plate morphology of the material (Figure 2b and panels a and b of Figure S4 of the Supporting Information). However, a detailed investigation performed in a wide region of the sample displayed larger aggregates of plate morphologies. Furthermore, the TEM images showed plate-like morphologies due to the enhanced π–π stacking within COF layers (Figure 2c and panels a and b of Figure S5 of the Supporting Information). The inherent porosity of **Tfpa-Od** was assessed using a N_2_ adsorption/desorption analysis at 77 K (Figure 2d). As per the International Union of Pure and Applied Chemistry (IUPAC) classification, **Tfpa-Od** exhibits a type II gas adsorption isotherm. The resulting Brunauer–Emmett–Teller (BET) surface area of **Tfpa-Od** was calculated to be 554.9 m^2^ g^–1^. As the experimental PXRD pattern of **Tfpa-Od** was found to match well with the PXRD pattern generated from the simulated ABC model, it results in a narrower pore size than the AA-stacking configuration. This phenomenon suggests the microporous nature of **Tfpa-Od**, a hypothesis further supported by the computation of pore size distribution curves (with an average pore size of 2.76 nm) derived from BET adsorption isotherm studies (Figure S7 of the Supporting Information). The presence of pores exceeding 3 nm in size within the distribution curve suggests the existence of mesopores in the material, albeit in negligible quantities relative to overall calculations.^33^

**Figure 3:**
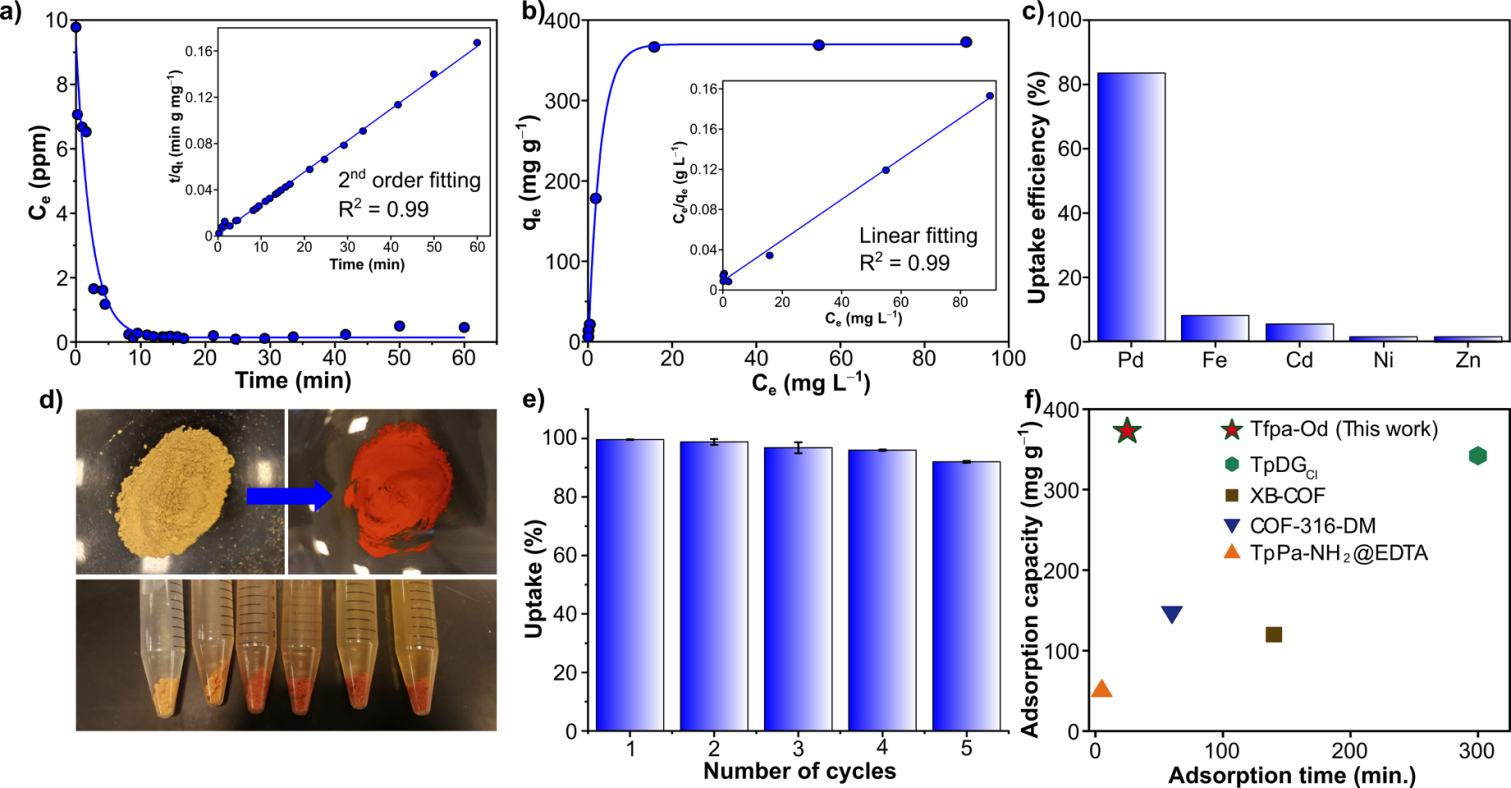
(a) Kinetics of Pd^2+^ adsorption toward **Tfpa-Od** and its pseudo-second-order fitting (inset), (b) adsorption isotherm of **Pd**^**2+**^**@Tfpa-Od** and adsorption isotherm fitting for the Langmuir linear adsorption equation (inset), (c) selectivity of Pd^2+^ adsorption toward **Tfpa-Od** compared to other divalent metal ions, (d) transition of color of **Tfpa-Od** with Pd^2+^ adsorption from bright yellow to brick red, (e) cyclic stability of **Tfpa-Od** toward Pd^2+^ adsorption, and (f) comparison of **Tfpa-Od** to other reported COF materials for Pd uptake.

Framework materials possessing electron-rich atoms, such as N, O, or S, are often considered excellent candidates for metal capture.^31,33^ Considering this, we explored the potential of **Tfpa-Od** for Pd^2+^ ion capture from an aqueous solution (Figure 1a). The batch adsorption kinetics was performed by continuously stirring 10 ppm of aqueous Pd^2+^ solution with 5 mg of **Tfpa-Od** (see section S2 of the Supporting Information). The ICP–MS results of the instantly collected residual solution samples showed fast adsorption kinetics with ∼90% Pd^2+^ removal within 5 min (Figure 3a). Notably, only 2.5% of metal remained in the residual solution after 10 min, whereas 100% removal efficiency was realized within 25 min of adsorption. The adsorption results exhibit a pseudo-second-order kinetics pathway, resulting in the rate constant of 0.006442 g mg^–1^ min^–1^ with a high correlation coefficient of >0.99 (inset in Figure 3a). In addition, the distribution coefficient (*K*_d_) for the material, which corresponds to the affinity of the absorbent to adsorbate, was found to be 3.62 × 10^6^ mL g^–1^. Observed *K*_d_ suggests an excellent affinity of Pd to the framework structure compared to recent literature concerning the sorbents.^25,31−34^

Furthermore, we investigated the thermodynamics of Pd^2+^ adsorption in water by treating **Tfpa-Od** with different Pd^2+^ concentrations ranging from 2 to 300 mg L^–1^ to obtain a saturation adsorption capacity (*q*_max_). The bright yellow color of **Tfpa-Od** turned dark brick red after adsorption of a high concentration of Pd^2+^ (Figure 3d). The obtained adsorption capacities (*q*_e_) at varied Pd^2+^ concentrations were further plotted with the equilibrium concentrations (*C*_e_), as shown in Figure 3b. The absorbent exhibited a saturated adsorption capacity of 372.59 mg g^–1^ (*q*_max_) for an initial Pd^2+^ concentration of 70 mg L^–1^, which is the highest reported for COF adsorbents in Pd^2+^ recovery (Figure 3f). Notably, the result is in good agreement with the Langmuir adsorption isotherm (*R*^2^ = 0.99), and the calculated adsorption constant *K*_a_ is found to be 0.2219 mg^–1^ L^–1^ (inset in Figure 3b). We further calculated *q*_max_ by the extrapolation of the linear Langmuir curve, i.e., *C*_e_/*q*_e_ versus *C*_e_, as shown in Figure 3b, and found the value of 395.26 mg g^–1^, which is in close agreement with the experimental observation. As the waste Pd^2+^ sources largely consist of other competing metal ions, testing the selectivity of the adsorbent material to ensure their quantitative removal is essential. Therefore, we investigated the selectivity of **Tfpa-Od** for Pd^2+^ ions over other interfering metals within industrial wastewater by preparing an aqueous solution containing 10 mg L^–1^ each of Fe^2+^, Cd^2+^, Ni^2+^, and Zn^2+^ (to relatively mimic the wastewater). When we performed the adsorption studies, a pronounced selective uptake of Pd^2+^ was observed with an efficiency of 83.2% within 25 min (Figure 3c and Figure S16 of the Supporting Information). In contrast, very poor uptake efficiency was observed for other metals: 7.8, 5.4, 1.4, and 1.7% uptake for Fe^2+^, Cd^2+^, Ni^2+^, and Zn^2+^ ions, respectively. For real-life applications, it is important to monitor the performance of adsorbent material in each successive adsorption/desorption cycle. In this direction, a continuous adsorption/desorption analysis for 5 mg of **Tfpa-Od** was performed by treating with 10 ppm of Pd^2+^ solution. After each adsorption study, the desorption was done by treating **Pd**^**2+**^**@Tfpa-Od** with stripping solution (0.2 M thiourea and 0.2 M HCl at pH 2) overnight in each cycle. It was observed that the material remained stable for up to 5 tested adsorption cycles with an average adsorption capacity of 332.15 mg g^–1^ (Figure 3e). In addition, there was no significant reduction in the crystallinity of the COF upon consecutive adsorption/desorption cycles (Figure S8 of the Supporting Information). To find the optimum pH for Pd uptake, we investigated the performance of Pd adsorption in different pH values ranging from 2 to 8 (Figure S17 of the Supporting Information). An adsorption efficiency of 88.41, 89.69, and 96.7% was observed for pH 5, 6, and 7, respectively, indicating the suitability of the material for Pd recovery in the optimum pH range of wastewater.

Furthermore, we evaluated the role of the hydrazone core of **Tfpa-Od** in metal ion uptake by carrying out a control imine COF synthesis. The synthesis involved the mixing of Tfpa (1 mol), 1,4-phenylenediamine (Pda, 1.5 mol), and acetic acid (0.3 mL, 6 M), followed by sonication in a pressure tube for 15 min under an inert atmosphere. Subsequently, the sealed tube was heated at 120 °C for 72 h to obtain the **Tfpa-Pda** COF. The crystallinity of **Tfpa-Pda** was determined by PXRD patterns (Figure S14 of the Supporting Information). The low-intensity first peak and broad peak corresponding to diffraction from 100 and 001 facets suggest the poor crystalline nature of the material. In addition, the experimental PXRD pattern agreed with the PXRD pattern simulated for the ABC conformation. The porosity of **Tfpa-Pda** was assessed through nitrogen gas adsorption isotherm measurements conducted at 77 K (Figure 2d). The resulting data revealed a type I BET isotherm, in accordance with IUPAC classification, with a surface area of 161 m^2^ g^–1^. Conversely, the BET isotherm of **Tfpa-Od** displayed a surface area of ∼555 m^2^ g^–1^. Pore size analysis was performed using non-local density functional theory modeling, revealing an almost equal average pore size of 2.76 nm for both **Tfpa-Pda** and **Tfpa-Od** COFs (Figure S15 of the Supporting Information). These findings indicate that hydrazone-based **Tfpa-Od** exhibits higher porosity than imine-based **Tfpa-Pda**. Additionally, to gain further insight into its adsorption performance at coordinative sites, **Tfpa-Pda** was evaluated for palladium removal, in which no adsorption was found (Figure S18 of the Supporting Information). This observation clearly suggests the strategic role of electron-rich hydrazone linkage toward quantitative Pd uptake by **Tfpa-Od**.

The **Pd**^**2+**^**@Tfpa-Od** sample was further characterized using various techniques. In the FTIR analysis, slight shifts were observed for peaks corresponding to C=O, C=N, and C–N to lower wave numbers, indicating the metal adsorption to the COF. The shift of the C–N absorption peak from 1175 to 1169 cm^–1^ further shows that the divalent Pd^2+^ ions can interact with N_amine_ of **Tfpa-Od** COF, besides their interaction with O_carbonyl_ (Figure S9 of the Supporting Information). In addition, the PXRD analysis showed sharp peaks at 2θ values, 40° and 46.65°, which correspond to 111 and 200 planes of Pd nanoparticles without much reduction of COF crystallinity, as shown in Figure S8 of the Supporting Information.^35,36^ To support this observation further, a detailed morphological analysis was carried out using SEM and TEM studies. **Pd**^**2+**^**@Tfpa-Od** displayed as aggregated microsized plate structures in SEM images (panels c and d of Figure S4 of the Supporting Information). In contrast, TEM images showed that the Pd nanoparticle fringes with the d-spacing values of 0.243 and 0.193 nm correspond to the 111 and 200 planes, respectively (panels c and d of Figure S5 and Figure S6 of the Supporting Information).^31,37^ The chemical environment of **Tfpa-Od** and **Pd**^**2+**^**@Tfpa-Od** was further studied using X-ray photoelectron spectroscopy (XPS) (panels e–l of Figure 2 and Figure S12 of the Supporting Information). An XPS survey spectrum for **Tfpa-Od** shows intense C 1s, N 1s, and O 1s peaks corresponding to carbon, nitrogen, and oxygen, respectively (Figure 2e).^31,33^ The survey spectrum for **Pd**^**2+**^**@Tfpa-Od** shows an additional peak at around 340 eV corresponding to Pd 3d, in addition to other peaks for elements present in the pristine COF. High-resolution XPS spectra for pristine and **Pd**^**2+**^**@Tfpa Od** samples are shown in panels f–l of Figure 2. The peaks located at 338.3 and 343.5 eV in the Pd 3d spectrum (Figure 2i) are attributed to the Pd 3d_5/2_ and Pd 3d_3/2_ peak splitting for Pd^2+^ in **Pd**^**2+**^**@Tfpa Od**. Figure 2i also shows that the partial reduction of Pd^2+^ ions to Pd nanoparticles upon adsorption occurred due to the presence of distinct peaks at more negative binding energies compared to the Pd 3d peaks for Pd^2+^, i.e., at 336.0 and 341.2 eV for Pd 3d_5/2_ and Pd 3d_3/2_, respectively.^38^ The coordinative interaction of Pd^2+^ with the hydrazone core of the **Tfpa-Od** framework is indicated by the shifts in binding energies of the deconvoluted peaks in N 1s and O 1s spectra (panels g and h of Figure 2 versus panels k and l of Figure 2). We infer that Pd is under coordination bond interaction with the O_carbonyl_ and N_amine_ atoms by forming an in-plane oriented five-membered ring (Figure 1a.). Also, considering the flexible nature of **Tfpa-Od**, as shown in Figure 1d, there could be a possibility for Pd to coordinate between N_amine_ of adjacent COF layers, which results in the partial reduction of divalent Pd ions adsorbed into Pd nanoparticles.^39^ The gas adsorption analysis of Pd2+@Tfpa-Od exhibited a type-II BET isotherm with a reduced surface area of 45.35 m2 g-1 upon Pd uptake (Figure S10). Furthermore, TGA suggests a thermal stability up to ∼300 °C. Notably, the slope of the curve after 300 °C was not as steep as TGA of pristine COF, indicating the stabilization of the COF after Pd^2+^ uptake (Figure S13b of the Supporting Information).

The constrained applicability of homogeneous catalytic reactions in industries, particularly concerning the effective retrieval of valuable Pd catalysts, promotes the utilization of heterogeneous solid catalysts. Therefore, employing heterogeneous COF-based Pd catalysts that can be recycled represents a promising remedy for this challenge. In this aspect, the catalytic activity of **Pd**^**2+**^**@Tfpa-Od** was investigated in one of the versatile Pd-catalyzed Suzuki–Miyaura coupling reactions. For that, a suspension of **Pd**^**2+**^**@Tfpa-Od** in methanol was treated with 0.5 M NaBH_4_ for 24 h to completely reduce Pd^2+^ into the Pd^0^ state (see section S2 of the Supporting Information). Subsequently, the sample was washed, dried, and isolated as a dark green powder of Pd nanoparticles over the **Tfpa-Od** (**Pd-NP@Tfpa-Od**) catalyst. The reduced Pd-adsorbed **Tfpa-Od** was further characterized via PXRD (Figure S8 of the Supporting Information). The TEM and SEM analyses showed much more aggregated stacking morphologies (panels e and f of Figures S4 and S5 of the Supporting Information). In addition, much more pronounced d spacing was observed for the Pd nanoparticles adsorbed in the TEM images corresponding to the 1 1 1 and 2 0 0 planes, as shown in Figure S6 of the Supporting Information.^31,37^ Additionally, the gas adsorption analysis of Pd-NP@Tfpa-Od displayed type-II BET isotherm with a surface area of 144.39 m2 g-1 (Figure S11 of the Supporting Information). Furthermore, a similar trend of mass stabilization was observed for the reduced **Pd-NP@Tfpa-Od** sample in TGA, further confirming the metalation-induced material stability (Figure S13c of the Supporting Information). The reduced COF catalyst was later used to analyze its catalytic potential in multiple substrate scopes with a range of substituted aryl iodides (0.5 mmol), with either electron-donating or electron-withdrawing groups (Table 1). For that, aryl iodides and phenylboronic acid (0.75 mmol) were treated with 0.5 mol % **Pd-NP@Tfpa-Od** catalyst in the presence of cesium carbonate (1 mmol) under an inert atmosphere. The reaction used 1,4-dioxane as the solvent for 24 h at 90 °C. As a preliminary observation, the reaction progress between iodobenzene and phenylboronic acid was monitored using gas chromatography–mass spectrometry (GC–MS). Analysis of the obtained product indicates the quantitative formation of biphenyl (yield of >99%; entry 1 in Table 1) with a retention time of 17.81 min (Figure S34 of the Supporting Information). Furthermore, several combinations of substituted aryl iodides were treated with phenylboronic acid to obtain substituted biaryls with calculated yields ranging from 79 to 99%. Details of Suzuki–Miyaura coupling reactions performed in this work are summarized in Table 1 and section S6 of the Supporting Information. The reaction of phenylboronic acid with aryl iodide consisting of electron-withdrawing groups (entries 2 and 6 in Table 1) gave >99% yield. Meanwhile, the reactions of aryl iodides substituted with electron-donating groups (entries 3, 4, and 5 in Table 1) afforded yields in the range of 79–91%. The recyclability of the Pd catalyst was evaluated through a reaction involving 1-iodo-4-nitrobenzene and phenylboronic acid, resulting in a consistent 96% conversion rate, even in the third cycle (Figures S32 and S35 and Table S4 of the Supporting Information). In addition, the reproduced catalyst was further treated with distilled water overnight, and the supernatant solution was tested using ICP–MS, further suggesting no considerable leaching of palladium metal even after completion of the catalytic cycle (<0.15%).

**Table 1:**
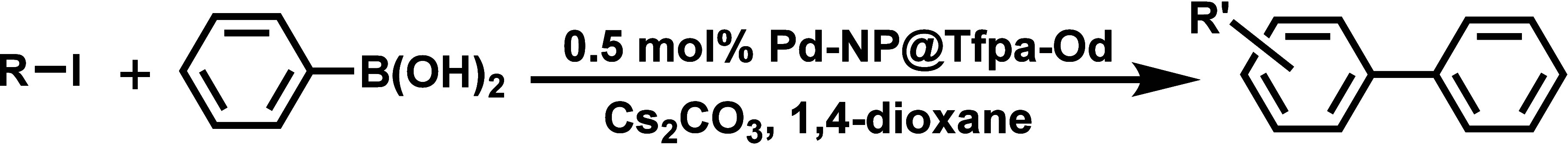

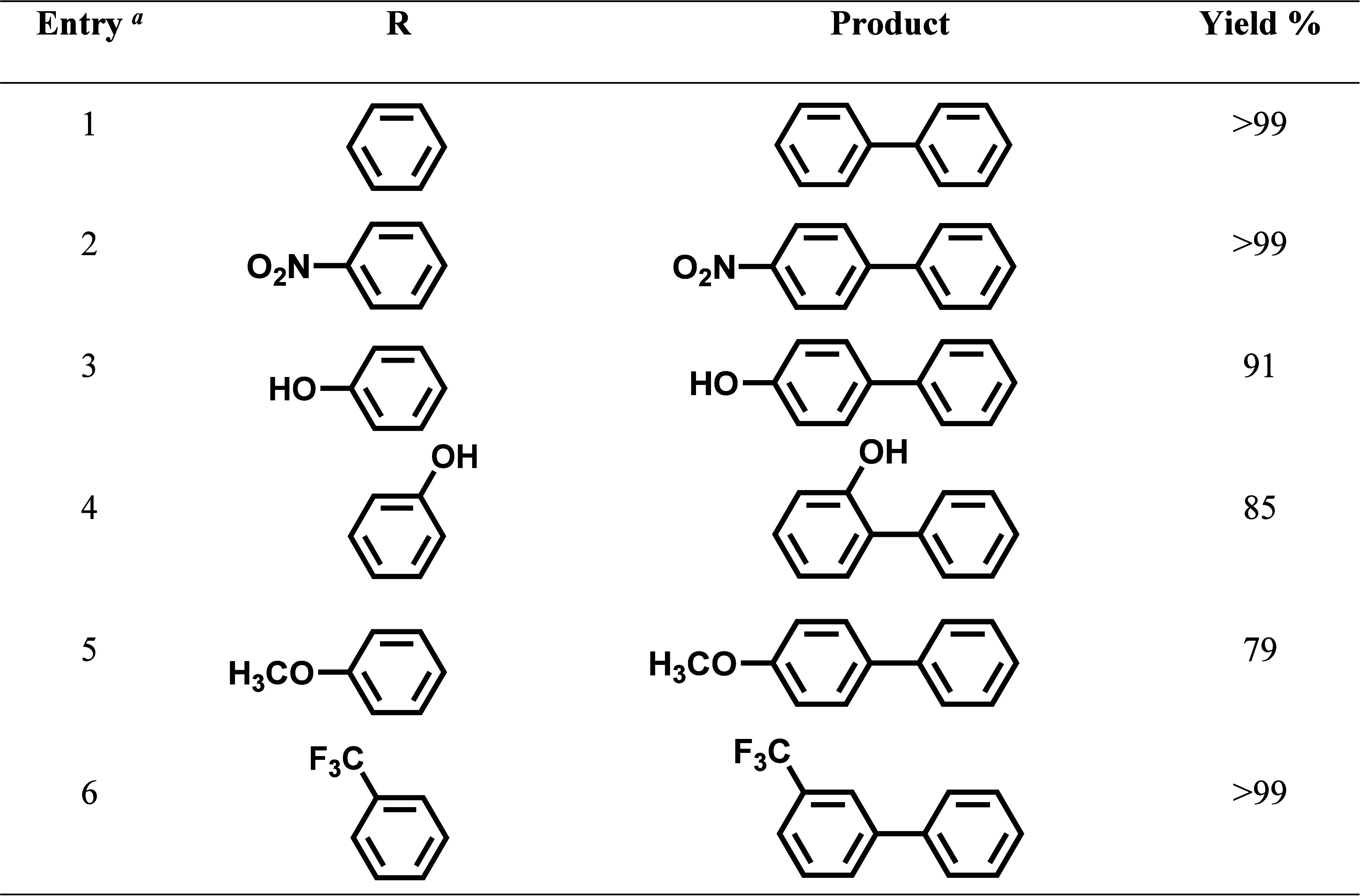
Catalytic Results Were Obtained When **Pd-NP@Tfpa-Od** Was Used for the Suzuki–Miyaura Coupling Reaction

a The reaction conditions are aryl halide (0.5 mmol), phenylboronic acid (0.75 mmol), Cs_2_CO_3_ (1.0 mmol), **Pd-NP@Tfpa-Od** (0.5 mol %), and 7 mL of 1,4-dioxane at 90 °C for 24 h.

## Conclusion

In summary, we synthesized a hydrazone-linked COF via a solvothermal reaction and explored its potential in the selective adsorption of Pd from wastewater. The semicrystalline mesoporous structure of **Tfpa-Od** COF provided well-exposed active sites within its two-dimensional (2D) pore channels. The numerous electron-rich anchoring sites made it an excellent choice for Pd^2+^ adsorption, resulting in the highest distribution coefficient and adsorption capacity reported for COF systems. Adsorbed **Pd-NP@Tfpa-Od** was further employed as a catalyst for the Suzuki–Miyaura coupling reaction, achieving a quantitative yield, even after 3 cycles. This research demonstrates the potential of COFs in the sustainable retrieval of precious metal ions from wastewater and the development of efficient heterogeneous COF-based catalysts.

## Supporting Information Available

Structural simulation, fractional atomic coordinates, additional PXRD patterns, FTIR spectra, SEM and TEM images, BET isotherms, XPS profiles, TGA plots, adsorption plots and calculations, and NMR data and figures (PDF)
Crystallographic information file of **Tfpa-Od** (CIF)
Crystallographic information file of **Tfpa-Pda** (CIF)

## Supplementary Material

am4c07706_si_001.pdf

am4c07706_si_002.cif

am4c07706_si_003.cif
