# Supplementary material for: Hydrazone-Linked Covalent Organic Framework Catalyst via Efficient Pd Recovery from Wastewater
Source: ACS Appl Mater Interfaces. 2024 Aug 21;17(12):17804–12. doi: 10.1021/acsami.4c07706 (PMC11956000; doi:10.1021/acsami.4c07706)
Supplement: Supplementary file 1 — am4c07706_si_001.pdf [file am4c07706_si_001.pdf]

Supporting Information

Hydrazone-Linked Covalent Organic Framework Catalyst *via* Efficient Pd-recovery from Wastewater

Mahira Bashri[a], Sushil Kumar[a], Pallab Bhandari[b], Sasi Stephen[a], Matthew J. O'Connor[c], Safa Gaber[a], Tina Škorjanc[d], Matjaž Finšgar[e], Gisha Elizabeth Luckachan[a], Blaž Belec[d], Emad Alhseinat[f], Partha Sarathi Mukherjee[b], Dinesh Shetty[a,g].*

[a]Department of Chemistry, Khalifa University of Science & Technology, P.O. Box 127788, Abu Dhabi, United Arab Emirates. E-mail: dinesh.shetty@ku.ac.ae

[b]Department of Inorganic and Physical Chemistry, Indian Institute of Science, Bangalore-560012, India.

[c]New York University Abu Dhabi, P.O. Box 129188, Abu Dhabi, United Arab Emirates.

[d]Materials Research Laboratory, University of Nova Gorica, Vipavska 11c, 5270 Ajdovscina, Slovenia.

[e]University of Maribor, Smetanova ulica 17, 2000 Maribor, Slovenia.

[f]Department of Chemical and Petroleum Engineering, Khalifa University of Science & Technology, P.O. Box 127788, Abu Dhabi, United Arab Emirates.

[g]Center for Catalysis& Separations (CeCaS), Khalifa University of Science & Technology, P.O. Box 127788, Abu Dhabi, United Arab Emirates.

Table of content

Title **Page No.**

Section S1. General Information……………………………………………………….………..3

S1.1. Materials.…………...…..………………………………...……….……….…...3

S1.2. Instrument techniques………………………………………………………......3

Section S2. Structural simulation and Pawley refinement……………………..………..…..6

Section S3. Material Characterizations………………………………….……………12

S3.1. PXRD studies……………………………………………………………....12

S3.2. FT-IR spectroscopic studies……………….…...……………….……………13

S3.3. Electron microscopy analysis……………….…………...…….……….…..14

S3.4. Gas adsorption studies…………………...…….…………….….………17

S3.5. Additional characterizations………………...…….…………….……….....…18

Section S4. Adsorption study………………………………...…………..……...………..24

S4.1. Additional experimental and calculation details…………...….…….……...…...…..24

S4.2. Additional adsorption results ……………………...…….….……….…………..25

Section S5. Catalytic study…………………………...………...………...…….………27

S5.1. NMR data and spectra….…………………………………………….………….27

S5.2. Additional results in catalytic study……….…….…..………….....…………...36

Section S6. Literature tables………………………..…...…………….………………..38

Section S7. References………………………...……...……………………….…………...40

Section S1. General Information

S1.1. Materials

All the chemicals were obtained commercially and used without further purification. 1,3,5-tris(4-formylphenyl)amine (Tfpa) (Sigma-Aldrich), mesitylene (Sigma-Aldrich), 1,4-dioxane (Sigma-Aldrich), thiourea (Sigma-Aldrich) acetic acid and hydrochloric acid (37%) (Merck-Supelco), Oxalyldihydrazide (Od) (TCI), Palladium(ll) Chloride (TCI), Sodium borohydride (Fischer Scientific), Milli-Q Water-18Ω (Merck-Millipore).

S1.2. Instrument techniques

Powder X-ray Diffraction (PXRD): Rigaku Smart Lab II with Cu K_α (λ = 1.5405 Å) radiation source operating at 40 kV and 40 mA was used for Powder X-ray diffraction measurement. The samples were analysed over the 2θ range of 2-50° with a step size of 0.02° (divergent slit=1/16°).

Fourier Transform Infrared (FT-IR): FT-IR spectra were taken on Bruker Optics ALPHA-E spectrometer with a universal Zn-Se attenuated total reflection (ATR) accessory in the 600-4000 cm^{-1} region or using a Diamond ATR (Golden Gate) with 32 scan rate and 4 cm^{-1} resolution.

Thermogravimetric Analysis (TGA): Perkin-Elmer Simultaneous ThermalanalyzerSTA 6000 under N_2 atmosphere was used for carrying out TGA at a heating rate of 10 °C min^{-1} within the 30-900 °C temperature range.

Scanning Electron Microscopy (SEM): SEM analysis of COF samples were analyzed using FEI Nova NanoSEM 650 consisting of an electron column with semi-in-lens detectors and an in-lens Schottky field emission gun to deliver ultra-high resolution with a wide range of probe current (1 pA to more than 200 nA). The images were recorded at a voltage of 3.5 keV. Samples for SEM analysis were prepared by drop-casting (~10 µL) COFs dispersed in isopropyl alcohol over a clean silicon substrate. Before the analysis, the dried samples were coated with Pt (nano-sized film) using JEOL JEC-300FC Auto Fine.

Transmission Electron Microscopy: TEM images were recorded using FEI Tecnai TEM 200 kV. Samples were prepared by drop casting COF powder dispersion in isopropyl alcohol over carbon-coated copper grids TEM Window (TED PELLA, INC. 200 mesh).

Nuclear Magnetic Resonance Spectroscopy (NMR): ^{13}Carbon Cross-Polarization Magic Angle Spinning (CP/MAS) solid-state NMR experiments were carried out using Bruker Avance NEO 500 MHz FT NMR spectrometer operating at a static field of 11.71 T, 4.0 mm MAS probe and standard linearly ramped cross-polarization (CP) pulse sequence. MAS probe was operated at a magic angle spinning rate of 12.0 kHz at ambient temperature. A CP pulse with contact time 2000 us, recycle delay of 2 sec and a total of 42200 scans was used for acquiring the spectra. ^{13}C chemical shifts were externally referenced to the adamantane CH_2 signal at 38.48 ppm. NMR data obtained was processed using TopSpin 4. 1. 4 software.

X-ray Photoelectron Spectroscopy (XPS): XPS analysis was performed using Supra$^+$ instrument (Kratos-Manchester, UK) equipped with an Al K_α source and a monochromator with a take-off angle of 90°. The charge neutralizer was turned on during the measurements, and data processing was performed using ESCApe 1.5 software (Kratos). The samples were placed over a silicon wafer attached to carbon tape. The analysis area was 300×700 microns with a pass energy of 20 eV and base pressure of the main analysis chamber at 2×10^{-9} mbar. Binding energy scale correction was done based on C-C/C-H peak at 284.8 eV in the C 1s spectra.

Gas Adsorption: Porosity analyses were performed using the Anton Paar Autosorb iQ combined physisorption and chemisorption instrument. 20-30 mg of COF samples were used for each analysis. Before doing N_2 gas adsorption in a liquid N2 bath (77K) for collecting full isotherm, the samples were activated by keeping them at 80 °C for 16 hours. The surface area was calculated using the multipoint Brunauer-Emmet-Teller (BET) model, whereas pore size distribution was found using the non-local density functional theory (NLDFT).

Inductively Coupled Plasma-Mass Spectrometry (ICP-MS): We employed two instruments for Pd quantification, which are as follows.

1. A Perkin Elmer NexION 2000 instrument was used for the elemental analyses. The high sensitivity and selectivity of the ICP-MS over other analytical techniques led to the choice of this instrument for Pd measurements. The following instrumental parameters were maintained during the analyses: RF Power: 1600W, Plasma gas flow: 14.5 L min^{-1}, Auxiliary gas flow: 1.2 L $min^{-1,}$ and Nebulizer gas flow: 0.96 l/min. The measurements were done in the kinetic energy discrimination (KED) mode with He as the collision gas. The instrument was tuned and optimized using the standard tune solutions provided by Perkin Elemer. Accordingly, the samples were

diluted to low levels before analyses; dilution is required so that the electron multiplier detector is not overloaded. As the LOQ of most metals including Pd is < 1.0 ppt (mg/L), even small variations in concentration could be detected and recorded.

2. The measurements were carried out with an Agilent 7800 ICP-MS instrument (ICP-MS/Agilent Technologies, Japan), equipped with a MicroMist nebulizer and a Peltier-cooled (2 ◦C) scott-type spray chamber for sample introduction. Ultrapure water (18MΩ cm resistivity) was obtained from an integral 10 milliQ water purification system (Millipore, Bedford, MA, USA). For the standard 4 element, Agilent ICP-MS tuning stock solution was used for tuning and calibration. Solutions were prepared from ICP-MS standard stocks for Pd (1000 mg mL–1, Sigma Aldrich) for quantitative determination. ICP-MS adjustment (RF power: 1550 W, nebulization gas flow: 1.05 L min^{-1}, Plasma Mode: General Purpose, Monitored Masses: ^{105}Pd) was performed daily using standard Agilent Calibration solution. A three-point calibration curve was created using the standard stock solutions of Pd for quantitative analysis (10 ppm, 50 ppm, and 100 ppm concentrations for each element).

Section S2. Structural simulation and Pawley refinement

Crystal structure models can be built with several different modellization software, such as Materials Studio, Schrödinger suite, Avogadro, etc. Typically, atomic coordinates are provided by the user, and then, geometrical optimization with the selected method (forcefield-based, DFT, DFTB+, etc) is completed. The atomic positions and cell parameters are optimized during this process based on energy minimization procedures.

The initial set of atomic coordinates can be obtained from previously reported structures or from scratch, using a topological approach commonly employed in reticular chemistry, where atoms are positioned according to the topology expected from the connectivity of the building blocks[1,2] In the case of covalent organic frameworks (COFs), the unit cell parameters, specifically the values of *a* and *b*are derived from the d-spacing value using Bragg's equation. This calculation utilizes the 2θ value of the first peak, corresponding to the diffraction from the (100) facet. The *c* value is determined from a peak typically found in the 18-25° range, corresponding to the diffraction from the (001) facet. For our study, considering the symmetry of the monomers used in COF preparation, the structure is modeled in the Triclinic P1 space group ($a = b \neq c$; $\alpha = \beta \neq \gamma$). Given the symmetry of the monomers and the formation of planar 2D organic sheets, COFs are typically modeled as layered structures. Three distinct stacking possibilities for these organic sheets are considered: 1) staggered AB and ABC arrangements, analogous to the packing of graphite sheets, where three-connected vertices (carbon atoms) align over the center of six-membered rings in neighboring graphite layers, and 2) eclipsed AA arrangements, where atoms of adjacent sheets align directly over each other, as seen in boron nitride. Each stacking model, staggered and eclipsed, exhibits unique diffraction patterns, as each space group has distinct symmetry-imposed reflection conditions. However, due to the limited number of experimental powder X-ray diffraction peaks often observed for COFs, refinement of atom positions in the simulated models using Rietveld methods is usually not feasible. Instead, a Pawley refinement method is commonly accepted to refine COF models. The degree of fitting for the refined profile, including unit cell parameters, is assessed using a difference plot and Rp values. This is the most commonly used approach in the field of COFs, where single crystals are usually not available[3-5]

After the optimization process, the final atomic coordinates, including space group symmetry and cell parameters, can be exported into a file with any format of choice. Among them, one can export

the information as a .cif file, and following this universally employed format facilitates the visualization of the structure.

Noting that the process of generating the atomic coordinates is not based on experimental data, such as in the case of single crystal diffraction, some of the information contained in the cif file differs from the one in cif files generated from refinement programs (i.e., no structure factors or refinement details are available). Conversely, the checkcif process, which is employed for experimentally determined crystal structures, cannot be applied with these files. The CIF file is provided here to facilitate the visualization and inspection of the proposed structure models generated from the modelization process, but it must not be treated as an experimentally determined crystal structure.

Analysis of the PXRD patterns suggests that the as-synthesized COF is crystalline. Two-dimensional modeling was performed using Materials Studio software to correlate the experimental PXRD pattern with the simulated one. Modeling was performed for three possible conformations, i.e., the AA, AB, and ABC stacking modes, using the triclinic (*P*1) space group. The experimental PXRD pattern agreed with the pattern simulated for the ABC conformation. The PXRD pattern was refined using the Pawley refinement in the Materials Studio software.

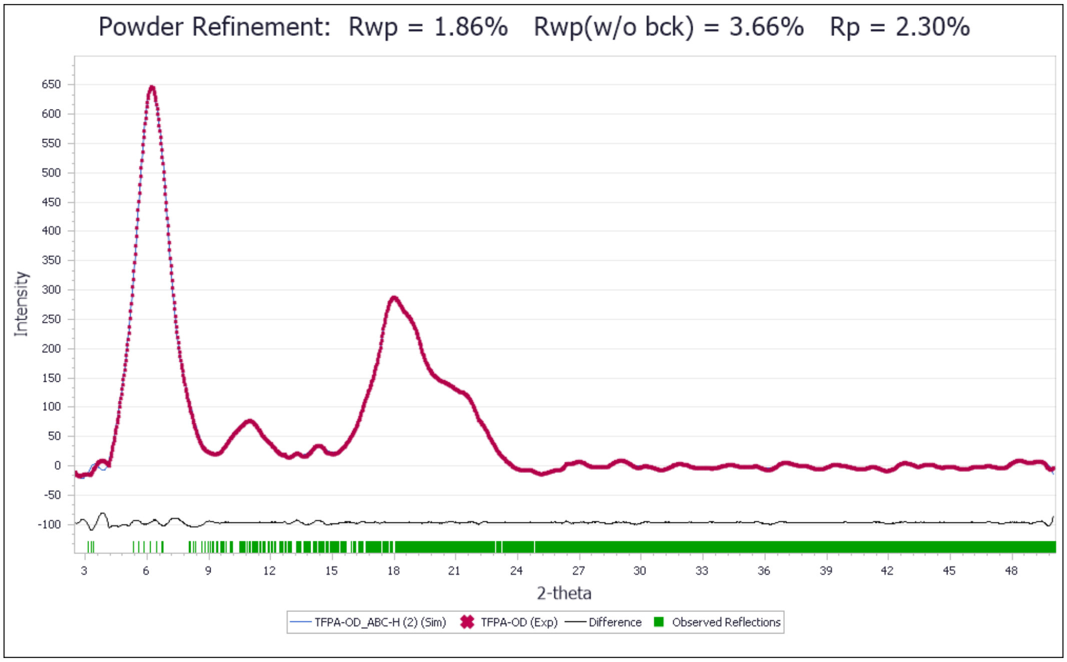

Figure S1. Pawley refinement of **Tfpa-Od**. After Pawley refinement, the experimental PXRD pattern is in agreement with the simulated pattern.

Table S1. Fractional atomic coordinates and lattice parameters of unit cell of **Tfpa-Od** for ABC model.

**TFPA-OD ABC model, Space group *P*1**											
**Unit cell parameters: a = 32.29 Å, b = 30.73 Å, c = 9.88 Å; α = 86.84°, β = 88.22°, γ = 115.25°**											
**Atoms**	**x**	**y**	**z**	**Atoms**	**x**	**y**	**z**	**Atoms**	**x**	**y**	**z**
**C1**	6.99545	1.95564	-0.12712	**C69**	0.12113	1.00673	0.39673	**C137**	0.02166	0.57361	0.12651
**C2**	6.94625	1.91761	-0.12847	**C70**	0.08526	1.0188	0.36917	**O138**	0.04402	0.61719	0.14899
**C3**	6.93396	1.86964	-0.14834	**N71**	-0.00221	0.99689	0.36759	**N139**	0.04558	0.54863	0.10271
**C4**	6.96996	1.85733	-0.17311	**C72**	0.03622	0.98689	0.40533	**N140**	0.09642	0.57111	0.11225
**C5**	7.01823	1.89452	-0.18478	**C73**	0.02538	0.94465	0.47937	**C141**	0.12002	0.55069	0.06404
**C6**	7.03037	1.94295	-0.16939	**C74**	0.0604	0.93111	0.49857	**C142**	0.1733	0.57725	0.0628
**C7**	6.95771	1.80899	-0.19125	**C75**	0.10874	0.96188	0.45717	**C143**	0.19784	0.58082	-0.06068
**N8**	6.91262	1.77533	-0.18452	**C76**	0.14397	0.9481	0.4732	**C144**	0.24817	0.61058	-0.06706
**N9**	6.89627	1.726	-0.20476	**N77**	0.13176	0.90465	0.51487	**C145**	0.27453	0.63539	0.05091
**C10**	6.84742	1.69458	-0.20783	**N78**	0.1651	0.88818	0.52849	**C146**	0.25024	0.62925	0.17645
**C11**	6.82783	1.64281	-0.23668	**C79**	0.14865	0.83979	0.54986	**C147**	0.19968	0.60105	0.18164
**O12**	6.81946	1.70973	-0.18714	**C80**	0.18297	0.82145	0.56473	**N148**	0.32541	0.67028	0.04105
**O13**	6.85535	1.62711	-0.25681	**O81**	0.10507	0.81132	0.55659	**C149**	0.34026	0.71956	-0.00006
**N14**	6.77886	1.61217	-0.24056	**O82**	0.22642	0.85026	0.57283	**C150**	0.36033	0.65805	0.09589
**N15**	6.76062	1.56272	-0.26376	**N83**	0.16658	0.77261	0.57015	**C151**	0.38659	0.74935	-0.05672
**C16**	6.71484	1.53151	-0.25543	**N84**	0.19954	0.756	0.59015	**C152**	0.40761	0.79978	-0.05257
**C17**	6.69893	1.47997	-0.26859	**C85**	0.18643	0.71026	0.59073	**C153**	0.38119	0.82071	0.00099
**C18**	6.65623	1.44562	-0.20461	**C86**	0.22329	0.69554	0.60612	**C154**	0.33193	0.79092	0.03197
**C19**	6.64511	1.39742	-0.19695	**C87**	0.21306	0.64908	0.5644	**C155**	0.31151	0.74054	0.03186
**C20**	6.67672	1.38251	-0.25337	**C88**	0.2499	0.63726	0.56186	**C156**	0.35292	0.61105	0.07912
**C21**	6.71596	1.41651	-0.33161	**C89**	0.29737	0.67177	0.60212	**C157**	0.3879	0.59995	0.12335
**C22**	6.72811	1.465	-0.33573	**C90**	0.30631	0.71679	0.65364	**C158**	0.4312	0.63575	0.18533
**N23**	6.66826	1.33338	-0.23655	**C91**	0.26988	0.72875	0.65472	**C159**	0.43827	0.68225	0.20592
**C24**	6.70649	1.32152	-0.21082	**N92**	0.33712	0.66261	0.58544	**C160**	0.40286	0.69308	0.16375
**C25**	6.6191	1.29379	-0.24601	**C93**	0.38519	0.70181	0.56058	**C161**	0.46961	0.62513	0.22328
**C26**	6.69368	1.27331	-0.17787	**C94**	0.32945	0.61442	0.58134	**N162**	0.46044	0.58089	0.21504
**C27**	6.72865	1.25974	-0.16511	**C95**	0.42425	0.70593	0.63817	**N163**	0.49508	0.5668	0.23627
**C28**	6.7778	1.29393	-0.18276	**C96**	0.47117	0.74275	0.60898	**C164**	0.48085	0.51902	0.21858
**C29**	6.7918	1.34254	-0.20246	**C97**	0.47936	0.77595	0.50172	**C165**	0.51642	0.50254	0.21252
**C30**	6.75678	1.35645	-0.21299	**C98**	0.44001	0.77352	0.43017	**O166**	0.43779	0.48951	0.20378
**C31**	6.58735	1.28566	-0.1382	**C99**	0.39311	0.73628	0.4586	**O167**	0.55921	0.53091	0.23604
**C32**	6.53976	1.24833	-0.14306	**C100**	0.32291	0.5907	0.45471	**N168**	0.50152	0.45549	0.17873
**C33**	6.5239	1.21522	-0.2526	**C101**	0.31444	0.54322	0.45186	**N169**	0.53508	0.43935	0.17465
**C34**	6.55732	1.21924	-0.35345	**C102**	0.31264	0.51949	0.57634	**C170**	0.5219	0.39417	0.15606
**C35**	6.60487	1.2588	-0.3506	**C103**	0.32176	0.54444	0.70194	**C171**	0.55848	0.37904	0.16161
**C36**	6.47228	1.1786	-0.26189	**C104**	0.33033	0.59181	0.70388	**C172**	0.54391	0.33006	0.17611
**N37**	6.44168	1.18927	-0.20753	**C105**	0.2964	0.46762	0.57922	**C173**	0.57841	0.31568	0.18981
**N38**	6.39135	1.15873	-0.20814	**N106**	0.26511	0.43925	0.4892	**C174**	0.62903	0.35003	0.19247
**C39**	6.36305	1.17719	-0.16427	**N107**	0.2427	0.38876	0.50026	**C175**	0.64277	0.39909	0.16916
**C40**	6.31039	1.14581	-0.14943	**C108**	0.20024	0.3599	0.43233	**C176**	0.60812	0.41335	0.15466
**O41**	6.38184	1.22013	-0.13411	**C109**	0.17463	0.30715	0.45374	**N177**	0.66635	0.33613	0.20936
**N42**	6.28284	1.16393	-0.09726	**O110**	0.18304	0.37797	0.35613	**C178**	0.71666	0.37394	0.22698
**O43**	6.29128	1.103	-0.18108	**O111**	0.1951	0.28877	0.51433	**C179**	0.65462	0.28545	0.21678
**N44**	6.23357	1.13222	-0.07223	**N112**	0.12839	0.27849	0.40685	**C180**	0.75134	0.38249	0.12641
**C45**	6.20487	1.14676	-0.02667	**N113**	0.10703	0.2282	0.42073	**C181**	0.8007	0.41333	0.15129
**C46**	6.15317	1.11152	-0.01989	**C114**	0.06537	0.19783	0.36919	**C182**	0.81567	0.43956	0.27522
**C47**	6.12037	1.12461	-0.0695	**C115**	0.05073	0.14601	0.37953	**C183**	0.78042	0.43847	0.36361
**C48**	6.07237	1.08939	-0.09196	**C116**	0.00881	0.04662	0.36189	**C184**	0.73103	0.40527	0.3403
**C49**	6.05573	1.03924	-0.06847	**C117**	0.03265	0.07513	0.24801	**C185**	0.61559	0.25149	0.29718
**C50**	6.0882	1.02826	0.00155	**C118**	0.0497	0.12469	0.24991	**C186**	0.59923	0.20192	0.28671
**C51**	6.13672	1.06351	0.0205	**C119**	0.52869	0.8102	0.45594	**C187**	0.62439	0.18535	0.20536

**N52**	6.00824	1.00329	-0.09521	**N120**	0.56382	0.80475	0.50082	**C188**	0.66714	0.21904	0.14043
**C53**	6.81405	1.27852	-0.18509	**N121**	0.61144	0.83277	0.45404	**C189**	0.68213	0.26848	0.14586
**N54**	6.79966	1.23302	-0.17017	**C122**	0.64342	0.81686	0.47694	**C190**	0.60441	0.13324	0.18014
**N55**	6.83062	1.21406	-0.18269	**C123**	0.69337	0.84579	0.42942	**N191**	0.55764	0.10527	0.18022
**C56**	6.81205	1.16552	-0.16352	**O124**	0.63031	0.77835	0.53586	**N192**	0.53239	0.0532	0.14596
**O57**	6.76966	1.13996	-0.12951	**N125**	0.72099	0.82495	0.40314	**C193**	0.47871	0.02547	0.10792
**C58**	6.84277	1.1439	-0.18251	**O126**	0.71048	0.8894	0.41238	**O194**	0.45567	0.0466	0.10454
**O59**	6.88259	1.16758	-0.23644	**N127**	0.76953	0.85614	0.37008	**C195**	0.40652	0.86977	0.05965
**N60**	6.82784	1.09811	-0.13679	**C128**	0.80063	0.84341	0.33425	**N196**	0.44742	0.88374	0.1235
**N61**	6.86019	1.0807	-0.14572	**C129**	0.85116	0.8826	0.31958	**C197**	0.86704	0.46441	0.31761
**C62**	6.85573	1.04469	-0.07349	**C130**	0.88897	0.87328	0.29298	**N198**	0.89467	0.44853	0.27029
**C63**	6.89457	1.03296	-0.0803	**C131**	0.93723	0.91125	0.30058	**N199**	0.47145	0.92679	0.21297
**C64**	6.89919	1.00513	0.02354	**C132**	0.94815	0.95826	0.34344	**C200**	0.52264	0.94551	0.26874
**C65**	6.93827	0.99683	0.0211	**C133**	0.90936	0.9675	0.35391	**O201**	0.54502	0.92537	0.23364
**C66**	6.97238	1.01503	-0.08592	**C134**	0.86202	0.93053	0.34254	**N202**	0.94502	0.46691	0.31687
**C67**	6.96702	1.04223	-0.18919	**C135**	0.00442	0.1155	0.46368	**C203**	0.97438	0.44362	0.27012
**C68**	6.92902	1.05181	-0.18684	**C136**	-0.00667	0.06614	0.46634	**O204**	0.95541	0.40788	0.19581
**H205**	6.91599	1.92356	-0.11478	**H241**	0.15833	1.03191	0.36642	**H277**	-0.01769	0.55456	0.12176
**H206**	6.89579	1.84226	-0.14394	**H242**	0.09654	1.0528	0.31721	**H278**	0.02555	0.5127	0.06949
**H207**	7.0466	1.88638	-0.2098	**H243**	-0.01081	0.92096	0.51907	**H279**	0.10111	0.51657	0.00913
**H208**	7.06735	1.97021	-0.19304	**H244**	0.04933	0.89632	0.54606	**H280**	0.1778	0.562	-0.1531
**H209**	6.98552	1.79972	-0.21332	**H245**	0.18133	0.97331	0.44518	**H281**	0.26601	0.61468	-0.16464
**H210**	6.92099	1.7138	-0.21935	**H246**	0.20228	0.91284	0.51932	**H282**	0.27047	0.64775	0.26839
**H211**	6.75483	1.62503	-0.2226	**H247**	0.12958	0.74755	0.55918	**H283**	0.18092	0.59776	0.27756
**H212**	6.68918	1.54298	-0.22674	**H248**	0.14893	0.68293	0.57209	**H284**	0.40822	0.73353	-0.08958
**H213**	6.63271	1.45643	-0.1542	**H249**	0.17719	0.62263	0.52812	**H285**	0.44579	0.82189	-0.07856
**H214**	6.61213	1.37354	-0.14367	**H250**	0.24163	0.60193	0.52367	**H286**	0.31085	0.80624	0.07327
**H215**	6.73791	1.40549	-0.38819	**H251**	0.34216	0.74358	0.68873	**H287**	0.27469	0.71863	0.06927
**H216**	6.76081	1.49115	-0.38873	**H252**	0.2786	0.76447	0.69065	**H288**	0.3207	0.58332	0.02771
**H217**	6.65651	1.24558	-0.15804	**H253**	0.41848	0.68058	0.72064	**H289**	0.38153	0.56364	0.10507
**H218**	6.717	1.22219	-0.14163	**H254**	0.50098	0.74448	0.66843	**H290**	0.47155	0.71041	0.25225
**H219**	6.8299	1.36983	-0.20906	**H255**	0.44575	0.79875	0.34718	**H291**	0.40966	0.7295	0.18094
**H220**	6.77052	1.39492	-0.21271	**H256**	0.363	0.73334	0.39823	**H292**	0.50547	0.65395	0.2524
**H221**	6.60019	1.30867	-0.05032	**H257**	0.32345	0.60866	0.35914	**H293**	0.5314	0.59276	0.25448
**H222**	6.51557	1.24518	-0.06016	**H258**	0.30835	0.52484	0.35326	**H294**	0.46485	0.43103	0.15907
**H223**	6.54647	1.1925	-0.43449	**H259**	0.32029	0.52675	0.79912	**H295**	0.48377	0.36726	0.14452
**H224**	6.63138	1.2613	-0.42475	**H260**	0.33733	0.61102	0.80058	**H296**	0.50576	0.30245	0.17591
**H225**	6.46009	1.14495	-0.31769	**H261**	0.3046	0.45239	0.66811	**H297**	0.56378	0.27711	0.18718
**H226**	6.37622	1.12218	-0.23535	**H262**	0.2562	0.37362	0.57075	**H298**	0.68049	0.42689	0.15982
**H227**	6.29821	1.20079	-0.07342	**H263**	0.10987	0.29328	0.35579	**H299**	0.62025	0.45132	0.13662
**H228**	6.21776	1.18507	-0.01158	**H264**	0.044	0.20972	0.31191	**H300**	0.73944	0.36469	0.02994
**H229**	6.1323	1.16183	-0.09826	**H265**	0.03875	0.05849	0.16146	**H301**	0.82696	0.41627	0.07479
**H230**	6.04984	1.10327	-0.12841	**H266**	0.069	0.14669	0.16117	**H302**	0.79174	0.46191	0.45258
**H231**	6.07613	0.99238	0.04284	**H267**	0.53487	0.83628	0.37512	**H303**	0.70342	0.40371	0.40798
**H232**	6.16158	1.05322	0.06489	**H268**	0.62171	0.86433	0.39671	**H304**	0.59611	0.26312	0.36369
**H233**	6.85173	1.30436	-0.20714	**H269**	0.70718	0.78753	0.41185	**H305**	0.56652	0.17653	0.34098
**H234**	6.86698	1.23674	-0.21018	**H270**	0.79032	0.80553	0.32492	**H306**	0.68761	0.20679	0.08018
**H235**	6.79456	1.07779	-0.08635	**H271**	0.88129	0.83681	0.26891	**H307**	0.71465	0.29207	0.09045
**H236**	6.82634	1.0266	-0.00008	**H272**	0.96578	0.90349	0.27532	**H308**	0.62823	0.11979	0.14735
**H237**	6.8727	0.99001	0.10572	**H273**	0.91422	1.00321	0.36808	**H309**	0.56021	0.03932	0.15502
**H238**	6.94063	0.9735	0.09595	**H274**	0.83386	0.93984	0.35668	**H310**	0.50642	0.03887	0.12722
**H239**	6.99407	1.05732	-0.26802	**H275**	0.01032	0.12965	0.57099	**H311**	0.38703	0.88968	0.06968
**H240**	6.92677	1.07399	-0.26807	**H276**	-0.03364	0.04126	0.53694	**H312**	0.88002	0.49152	0.399
**C1**	5.99545	0.95564	-0.12712	**N71**	-0.00221	-0.00311	0.36759	**H313**	0.44376	0.94028	0.23135
**N52**	7.00824	1.00329	-0.09521	**N71**	0.99779	0.99689	0.36759	**H314**	0.49619	0.93702	0.24221
**N52**	7.00824	2.00329	-0.09521	**C116**	0.00881	1.04662	0.36189	**H315**	0.95675	0.50008	0.39209
**C66**	5.97238	1.01503	-0.08592	**C132**	-0.05185	0.95826	0.34344	**H316**	0.96051	0.45672	0.29643

Table S2. Fractional atomic coordinates and unit cell parameter of **Tfpa-Od** AA model**.**

TFPA-OD AA model (space group *P*1)											
**Unit cell parameters: a = 32.53 Å, b = 33.02 Å, c = 4.51 Å; α = 96.15°, β = 83.01°, γ = 123.07°**											
**Atoms**	**x**	**y**	**z**	**Atoms**	**x**	**y**	**z**	**Atoms**	**x**	**y**	**z**
**C1**	1.02188	-0.48576	-0.36261	**C37**	1.58121	-0.68403	-1.02289	**H73**	1.26968	-0.32092	-0.30367
**O2**	1.0363	-0.44294	-0.35346	**C38**	1.63114	-0.65087	-0.96604	**H74**	1.18245	-0.38088	-0.33254
**N3**	1.05264	-0.49922	-0.29767	**C39**	1.64841	-0.60109	-0.9521	**H75**	1.41848	-0.26125	-0.40343
**N4**	1.10193	-0.46385	-0.24832	**C40**	1.6167	-0.58549	-0.97095	**H76**	1.44864	-0.17559	-0.38746
**C5**	1.13113	-0.47426	-0.15538	**N41**	1.66381	-0.66745	-0.93044	**H77**	1.33316	-0.19875	0.28993
**C6**	1.18347	-0.43648	-0.12377	**C42**	1.71222	-0.63506	-0.81888	**H78**	1.30212	-0.28515	0.26406
**C7**	1.21333	-0.44713	0.00579	**C43**	1.64443	-0.71866	-0.93666	**H79**	1.32906	-0.40576	-0.47311
**C8**	1.26359	-0.41253	0.02169	**C44**	1.75342	-0.63627	-0.94409	**H80**	1.38603	-0.43299	-0.56305
**C9**	1.28521	-0.36662	-0.09399	**C45**	1.79961	-0.60511	-0.83215	**H81**	1.48087	-0.32166	0.06645
**C10**	1.25469	-0.35584	-0.2161	**C46**	1.80585	-0.57146	-0.59553	**H82**	1.42387	-0.29454	0.15286
**C11**	1.20451	-0.39024	-0.23111	**C47**	1.76507	-0.57023	-0.46933	**H83**	1.50592	-0.37561	-0.18066
**N12**	1.33797	-0.33185	-0.09728	**C48**	1.71877	-0.60251	-0.57497	**H84**	1.53126	-0.42662	-0.42018
**C13**	1.35754	-0.28031	-0.07307	**C49**	1.60809	-0.74741	-0.71804	**H85**	1.47777	-0.5664	-0.99036
**C14**	1.37154	-0.34837	-0.1492	**C50**	1.5893	-0.79686	-0.71845	**H86**	1.49551	-0.62899	-1.08583
**C15**	1.39942	-0.24822	-0.25237	**C51**	1.60788	-0.81819	-0.93259	**H87**	1.51146	-0.69481	-1.08424
**C16**	1.41679	-0.19904	-0.24197	**C52**	1.64394	-0.7896	-1.15286	**H88**	1.56574	-0.72249	-1.04866
**C17**	1.39293	-0.18061	-0.04848	**C53**	1.66134	-0.74049	-1.15845	**H89**	1.68683	-0.57348	-0.93388
**C18**	1.35203	-0.21214	0.13774	**C54**	1.59065	-0.86976	-0.92815	**H90**	1.63141	-0.54702	-0.95391
**C19**	1.3344	-0.26149	0.12436	**N55**	1.55897	-0.89662	-0.71946	**H91**	1.75102	-0.65984	-1.13437
**C20**	1.36183	-0.38741	-0.3527	**N56**	1.54311	-0.94544	-0.7069	**H92**	1.83065	-0.6065	-0.93612
**C21**	1.39452	-0.40291	-0.40431	**C57**	1.50892	-0.97306	-0.48703	**H93**	1.76887	-0.54479	-0.28518
**C22**	1.43779	-0.37946	-0.25376	**O58**	1.49131	-0.9554	-0.3053	**H94**	1.68793	-0.60118	-0.4728
**C23**	1.44772	-0.34037	-0.05214	**C59**	1.40944	-0.12912	-0.04387	**H95**	1.59423	-0.73132	-0.54788
**C24**	1.41504	-0.32496	-0.00122	**N60**	1.44295	-0.10087	-0.24082	**H96**	1.56114	-0.81802	-0.5468
**C25**	1.47314	-0.39477	-0.30342	**C61**	1.85441	-0.53695	-0.48045	**H97**	1.65808	-0.80546	-1.3242
**N26**	1.46569	-0.43056	-0.48911	**N62**	1.89015	-0.54401	-0.53572	**H98**	1.68702	-0.72005	-1.34075
**N27**	1.49931	-0.44521	-0.53539	**N63**	1.45849	-0.0521	-0.25022	**H99**	1.60546	-0.88502	-1.09964
**C28**	1.48997	-0.48437	-0.72175	**C64**	1.49302	-0.02417	-0.46778	**H100**	1.55781	-0.96058	-0.8658
**C29**	1.52471	-0.50063	-0.76034	**O65**	1.51088	-0.04166	-0.64883	**H101**	1.39229	-0.1153	0.12049
**O30**	1.45253	-0.50633	-0.85442	**N66**	1.93683	-0.5107	-0.43241	**H102**	1.85973	-0.5058	-0.34133
**O31**	1.56478	-0.47527	-0.65725	**C67**	1.97143	-0.52236	-0.44598	**H103**	1.4431	-0.03741	-0.09297
**N32**	1.51224	-0.54406	-0.90432	**O68**	1.96069	-0.56319	-0.5287	**H104**	1.94522	-0.47659	-0.34487
**N33**	1.54472	-0.5599	-0.9208	**H69**	1.03974	-0.53589	-0.28976	**C1**	2.02188	-0.48576	-0.36261
**C34**	1.53235	-0.60324	-1.01769	**H70**	1.1173	-0.51114	-0.10435	**C57**	1.50892	0.02694	-0.48703
**C35**	1.56686	-0.61902	-1.01347	**H71**	1.19786	-0.48245	0.09181	**C64**	1.49302	-1.02417	-0.46778
**C36**	1.54968	-0.66827	-1.04624	**H72**	1.28575	-0.42206	0.11883	**C67**	0.97143	-0.52236	-0.44598

Table S3. Fractional atomic coordinates and unit cell parameters of **Tfpa-Od** for AB model.

TFPA-OD AB model (space group *P*1)											
**Unit cell parameters: a = 33.83 Å, b = 33.55 Å, c = 6.29 Å; α = 89.99°, β = 90°, γ = 118.38°**											
**Atoms**	**x**	**y**	**z**	**Atoms**	**x**	**y**	**z**	**Atoms**	**x**	**y**	**z**
**N1**	5.99837	1.99923	-0.21704	**H141**	6.1791	1.97087	-0.21704	**C105**	0.58319	0.31798	-0.66652
**C2**	6.03737	1.98654	-0.21704	**H142**	6.19838	1.91056	-0.21704	**C106**	0.6313	0.34809	-0.66652
**C3**	6.03092	1.94112	-0.21704	**H143**	6.1344	1.75564	-0.21706	**C107**	0.63794	0.39369	-0.66652
**C4**	6.06413	1.92771	-0.21704	**H144**	6.15356	1.69532	-0.21752	**C108**	0.60478	0.40689	-0.66652
**C5**	6.10894	1.95913	-0.21704	**H145**	6.17711	1.63748	-0.2161	**N109**	0.66911	0.33431	-0.66652
**C6**	6.11996	2.0039	-0.21704	**H146**	6.22535	1.61629	-0.21446	**C110**	0.71949	0.3713	-0.66652
**C7**	6.08607	2.01681	-0.21704	**H147**	6.32939	1.75649	-0.21439	**C111**	0.65628	0.28345	-0.66652
**C8**	6.14396	1.94535	-0.21704	**H148**	6.27783	1.77497	-0.21641	**C112**	0.75852	0.36379	-0.66652
**N9**	6.13255	1.90274	-0.21704	**H149**	6.42683	1.66734	-0.21463	**C113**	0.80319	0.39745	-0.66652
**N10**	6.16418	1.88715	-0.21704	**H150**	6.4948	1.71834	-0.21603	**C114**	0.81334	0.44258	-0.66653
**C11**	6.15006	1.84167	-0.21703	**H151**	6.45774	1.82232	-0.21572	**C115**	0.78031	0.45369	-0.66652

**C12**	6.18282	1.82458	-0.21707	**H152**	6.38895	1.7747	-0.21465	**C116**	0.73604	0.42	-0.66652
**O13**	6.10994	1.81489	-0.21704	**H153**	6.24154	1.57259	-0.21462	**C117**	0.61126	0.24564	-0.66652
**O14**	6.22298	1.85128	-0.21705	**H154**	6.22461	1.50521	-0.2155	**C118**	0.60029	0.20032	-0.66652
**N15**	6.16857	1.7791	-0.21726	**H155**	6.36195	1.53768	-0.21648	**C119**	0.63372	0.18771	-0.66652
**N16**	6.20012	1.7635	-0.21745	**H156**	6.38267	1.60897	-0.21468	**C120**	0.67704	0.22017	-0.66652
**C17**	6.18865	1.72093	-0.21769	**H157**	6.30295	1.4561	-0.21757	**C121**	0.68746	0.26544	-0.66652
**C18**	6.22368	1.70745	-0.21462	**H158**	6.24272	1.37765	-0.21708	**C122**	0.62257	0.14027	-0.66652
**C19**	6.21273	1.66282	-0.21215	**H159**	6.08779	1.28862	-0.21704	**N123**	0.58009	0.11283	-0.66652
**C20**	6.24663	1.65021	-0.20787	**H160**	6.02765	1.21008	-0.21704	**N124**	0.55623	0.06424	-0.66652
**C21**	6.29503	1.6802	-0.20705	**H161**	5.96851	1.12857	-0.21704	**C125**	0.5057	0.04612	-0.66652
**C22**	6.30168	1.72589	-0.20804	**H162**	5.9468	1.05736	-0.21704	**O126**	0.49374	0.0752	-0.66652
**C23**	6.26836	1.73909	-0.21239	**H163**	6.0876	1.09158	-0.21704	**C127**	0.38296	0.86039	-0.66579
**N24**	6.33303	1.66627	-0.20757	**H164**	6.10549	1.15996	-0.21704	**N128**	0.42546	0.88784	-0.66573
**C25**	6.38425	1.70371	-0.20666	**H165**	6.53881	1.84482	-0.21753	**C129**	0.85843	0.47886	-0.66652
**C26**	6.31888	1.61444	-0.20669	**H166**	6.6179	1.86391	-0.21709	**N130**	0.88852	0.46642	-0.66645
**C27**	6.42309	1.69586	-0.20773	**H167**	6.71275	1.80014	-0.21704	**N131**	0.44931	0.93645	-0.6663
**C28**	6.46884	1.72877	-0.21162	**H168**	6.79196	1.81939	-0.21704	**C132**	0.49985	0.95462	-0.66583
**C29**	6.48202	1.77438	-0.21371	**H169**	6.87312	1.84163	-0.21704	**O133**	0.51186	0.92558	-0.66672
**C30**	6.44832	1.7863	-0.21129	**H170**	6.94219	1.8885	-0.21704	**N134**	0.93514	0.49458	-0.66634
**C31**	6.40252	1.75306	-0.2074	**H171**	6.90658	1.99738	-0.21704	**C135**	0.9589	0.46643	-0.66919
**C32**	6.27333	1.57679	-0.207	**H172**	6.83729	1.94653	-0.21704	**O136**	0.93536	0.42539	-0.66538
**C33**	6.26017	1.53044	-0.21106	**N1**	5.99837	0.99923	-0.21704	**H173**	0.04465	0.50711	-0.66498
**C34**	6.29137	1.51555	-0.21401	**N1**	6.99837	1.99923	-0.21704	**H174**	0.06425	0.46862	-0.66654
**C35**	6.33605	1.54813	-0.21226	**C50**	6.01125	2.05092	-0.21704	**H175**	0.13901	0.48624	-0.66595
**C36**	6.34868	1.59468	-0.20817	**C66**	5.94742	1.96066	-0.21704	**H176**	0.21523	0.51067	-0.66824
**C37**	6.27709	1.4664	-0.21748	**C69**	0.04646	0.53409	-0.66993	**H177**	0.281	0.55745	-0.66889
**N38**	6.23448	1.43636	-0.2173	**O70**	0.06995	0.57512	-0.66506	**H178**	0.24094	0.6641	-0.66886
**N39**	6.21896	1.38913	-0.21731	**N71**	0.0703	0.50601	-0.666	**H179**	0.17526	0.61258	-0.66756
**C40**	6.17354	1.35838	-0.2171	**N72**	0.11694	0.53419	-0.66562	**H180**	0.42643	0.75996	-0.66923
**C41**	6.15695	1.30799	-0.21704	**C73**	0.14702	0.52174	-0.66556	**H181**	0.44075	0.82611	-0.66797
**O42**	6.14617	1.37233	-0.21705	**C74**	0.19211	0.55801	-0.66957	**H182**	0.3026	0.79104	-0.66733
**O43**	6.18439	1.29415	-0.21704	**C75**	0.22513	0.5469	-0.67294	**H183**	0.2844	0.72213	-0.66874
**N44**	6.11154	1.27712	-0.21704	**C76**	0.26938	0.58061	-0.67698	**H184**	0.34021	0.57625	-0.66888
**N45**	6.09607	1.22983	-0.21704	**C77**	0.28593	0.62929	-0.67716	**H185**	0.39106	0.55777	-0.66705
**C46**	6.05344	1.19967	-0.21704	**C78**	0.24692	0.63678	-0.67604	**H186**	0.49148	0.69553	-0.66753
**C47**	6.03891	1.15027	-0.21704	**C79**	0.20226	0.60313	-0.67189	**H187**	0.44294	0.71647	-0.66892
**C48**	5.99414	1.11768	-0.21704	**N80**	0.33631	0.66629	-0.67587	**H188**	0.51522	0.63782	-0.66605
**C49**	5.98117	1.07085	-0.21704	**C81**	0.34916	0.71716	-0.67691	**H189**	0.53461	0.57768	-0.6665
**C50**	6.01125	1.05092	-0.21704	**C82**	0.37409	0.65249	-0.67644	**H190**	0.47074	0.42288	-0.66652
**C51**	6.05654	1.08862	-0.21704	**C83**	0.39417	0.75497	-0.67636	**H191**	0.49012	0.36274	-0.66652
**C52**	6.06988	1.13504	-0.21704	**C84**	0.40517	0.80029	-0.67212	**H192**	0.51386	0.30504	-0.66652
**C53**	6.53033	1.80925	-0.21734	**C85**	0.37176	0.81292	-0.66935	**H193**	0.56235	0.28404	-0.66652
**N54**	6.56209	1.79763	-0.21723	**C86**	0.32844	0.78047	-0.67179	**H194**	0.66519	0.42439	-0.66652
**N55**	6.60832	1.82934	-0.21733	**C87**	0.31802	0.7352	-0.67604	**H195**	0.61427	0.44281	-0.66652
**C56**	6.64067	1.8153	-0.2171	**C88**	0.36745	0.60691	-0.67539	**H196**	0.76455	0.33647	-0.66652
**C57**	6.6899	1.84863	-0.21704	**C89**	0.40058	0.59369	-0.67104	**H197**	0.83022	0.38801	-0.66652
**O58**	6.62901	1.77485	-0.21705	**C90**	0.44514	0.62544	-0.66894	**H198**	0.79024	0.48993	-0.66652
**N59**	6.72238	1.83474	-0.21704	**C91**	0.45601	0.67006	-0.67155	**H199**	0.72442	0.44319	-0.66652
**O60**	6.70143	1.88904	-0.21704	**C92**	0.42217	0.68257	-0.67579	**H200**	0.57895	0.2406	-0.66652
**N61**	6.76869	1.86654	-0.21704	**C93**	0.48019	0.61213	-0.66586	**H201**	0.5647	0.17447	-0.66652
**C62**	6.80057	1.85501	-0.21704	**N94**	0.46881	0.56961	-0.66611	**H202**	0.70287	0.20956	-0.66652
**C63**	6.8492	1.88985	-0.21704	**N95**	0.50045	0.55417	-0.66631	**H203**	0.72112	0.27843	-0.66652
**C64**	6.88292	1.87776	-0.21704	**C96**	0.48628	0.50875	-0.6665	**H204**	0.64834	0.12945	-0.66652
**C65**	6.92903	1.91093	-0.21704	**C97**	0.51906	0.49182	-0.66654	**H205**	0.58486	0.05366	-0.66652
**C66**	6.94742	1.96066	-0.21704	**O98**	0.44615	0.48196	-0.66651	**H206**	0.53112	0.04622	-0.66652
**C67**	6.9087	1.9683	-0.21704	**O99**	0.5592	0.5186	-0.66652	**H207**	0.35721	0.87123	-0.6661
**C68**	6.86284	1.9356	-0.21704	**N100**	0.5049	0.44639	-0.66653	**H208**	0.86644	0.51435	-0.66649
**H137**	6.00267	1.91139	-0.21704	**N101**	0.53653	0.43095	-0.66652	**H209**	0.42069	0.94705	-0.66643
**H138**	6.05431	1.89178	-0.21704	**C102**	0.52515	0.38843	-0.66652	**H210**	0.47442	0.95444	-0.66701

**H139**	6.15568	2.02905	-0.21704	**C103**	0.5602	0.37512	-0.66652	**H211**	0.9412	0.53197	-0.66655
**H140**	6.1081	2.05105	-0.21704	**C104**	0.54935	0.3305	-0.66652	**H212**	0.96086	0.49351	-0.66521

Section S3. Material Characterizations

S3.1. PXRD studies

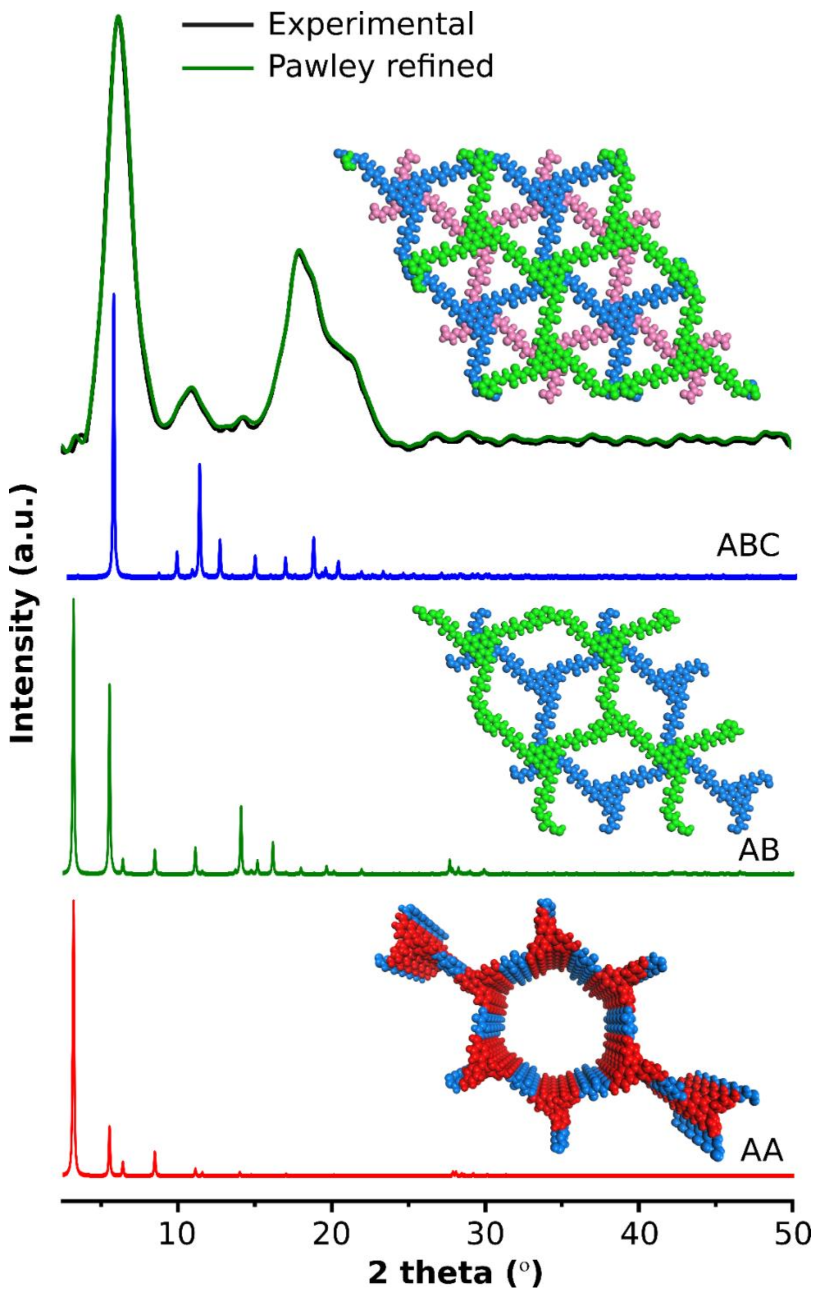

Figure S2. Comparison of **Tfpa-Od** PXRD to the simulated AA, AB and ABC model.

S3.2. FT-IR spectroscopic studies

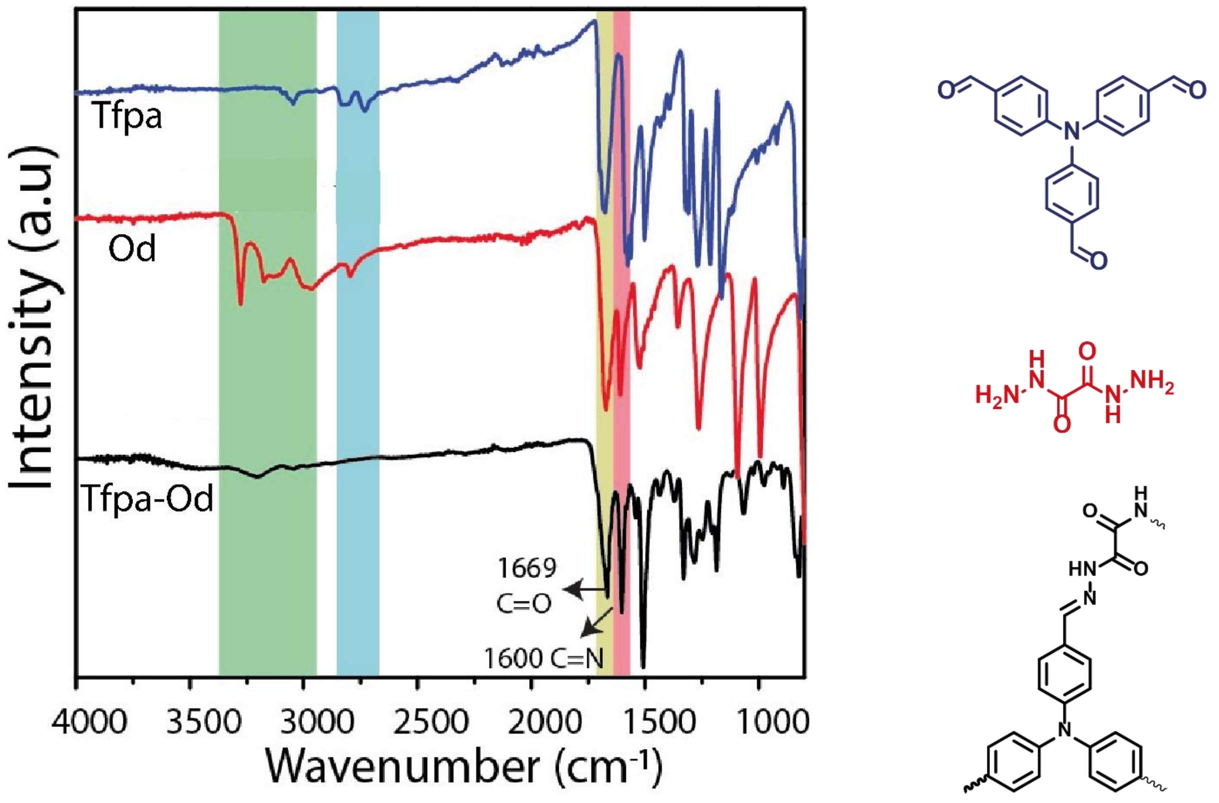

Figure S3. FT-IR spectra of **Tfpa-Od** COF when compared with monomers Tfpa and Od. Representative peaks are highlighted.

S3.3. Electron microscopy analysis

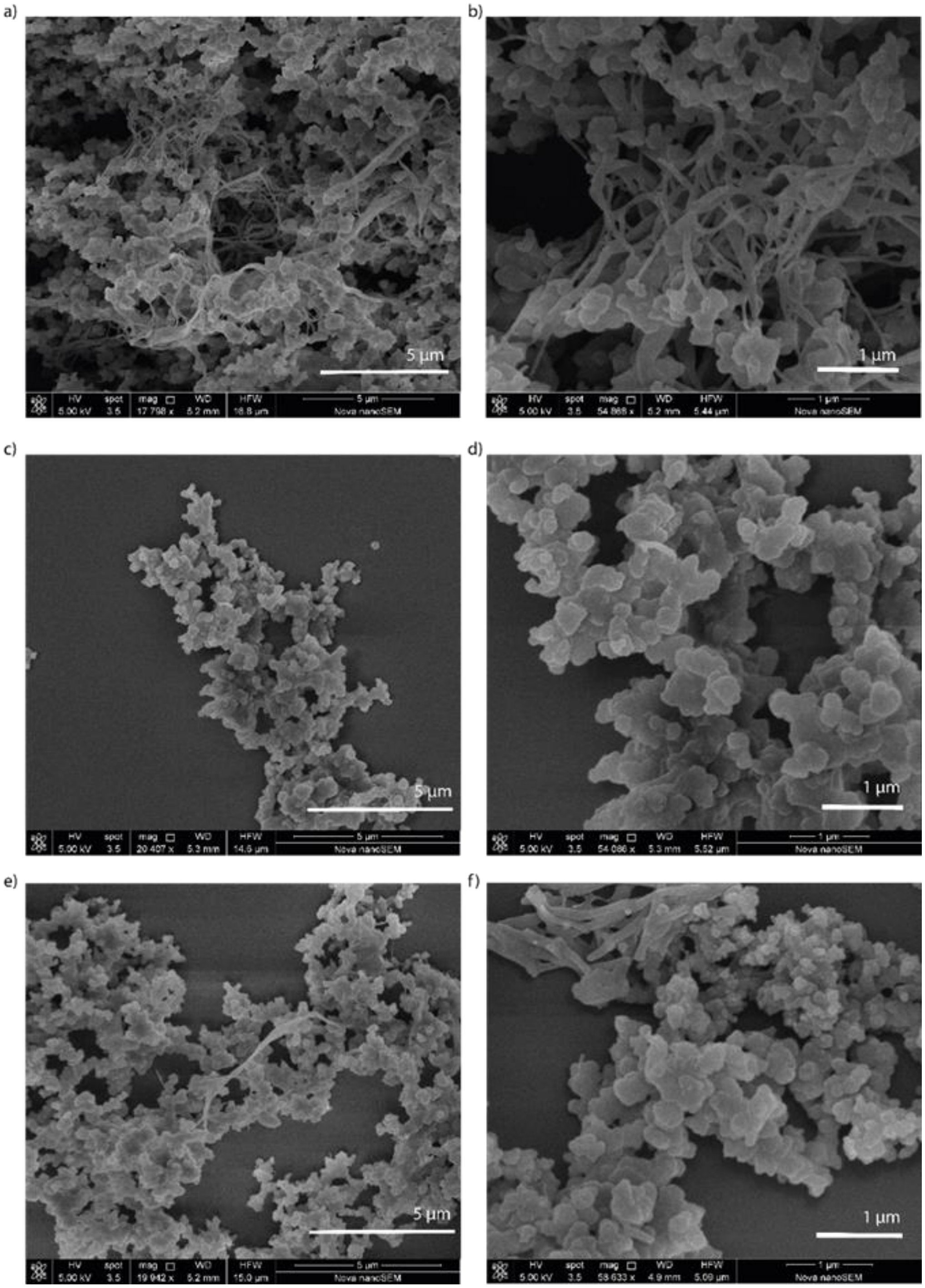

Figure S4. SEM images of **a, b) Tfpa-Od** COF**, c,d) Pd^{2+}@Tfpa-Od**, and e,f) **Pd-NP@Tfpa-Od.**

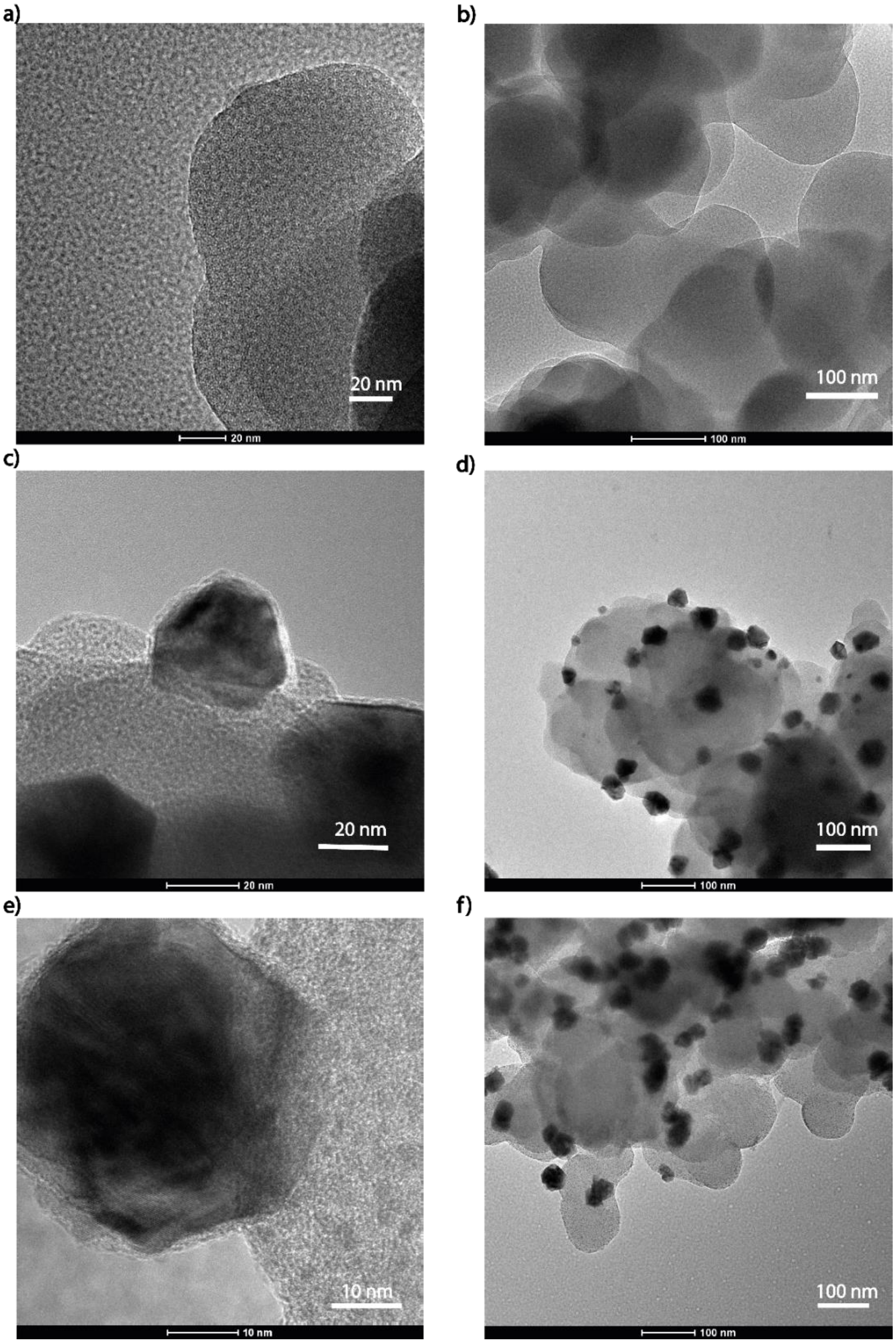

Figure S5. TEM images of a,b) **Tfpa-Od** COF, c,d) **Pd^{2+}@Tfpa-**Od, e,f) **Pd-NP@Tfpa-Od.**

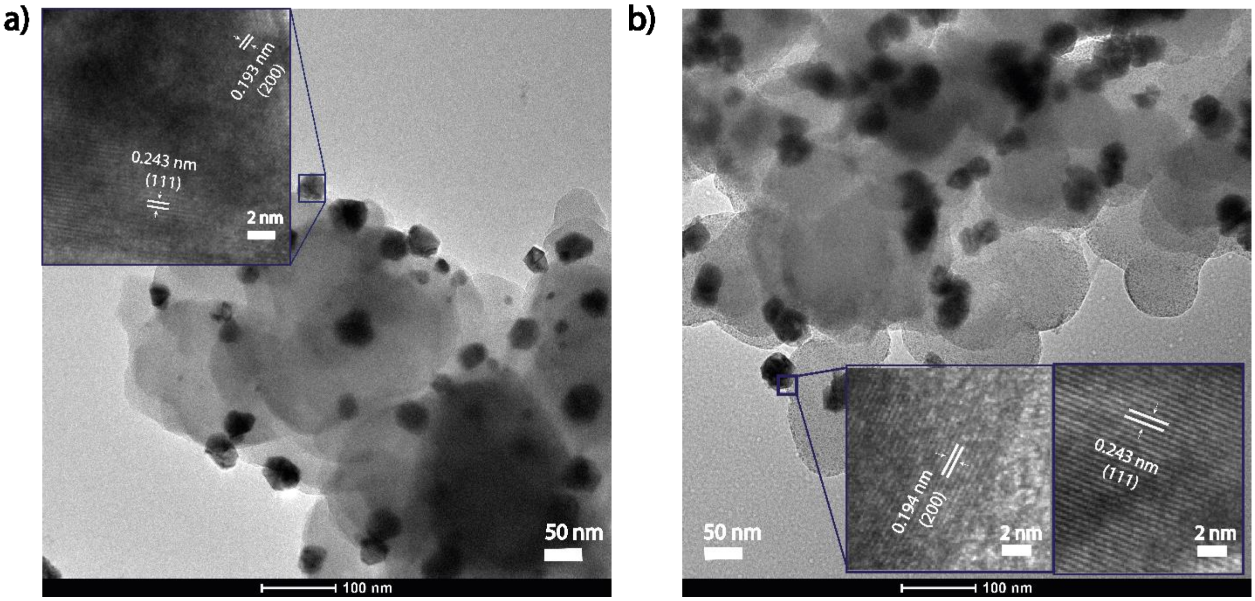

Figure S6. TEM fringes corresponds to Pd (111) and (200) planes in a) **Pd^{2+}@Tfpa-Od** and b) **Pd-NP@Tfpa-Od.**

S3.4. Gas adsorption studies

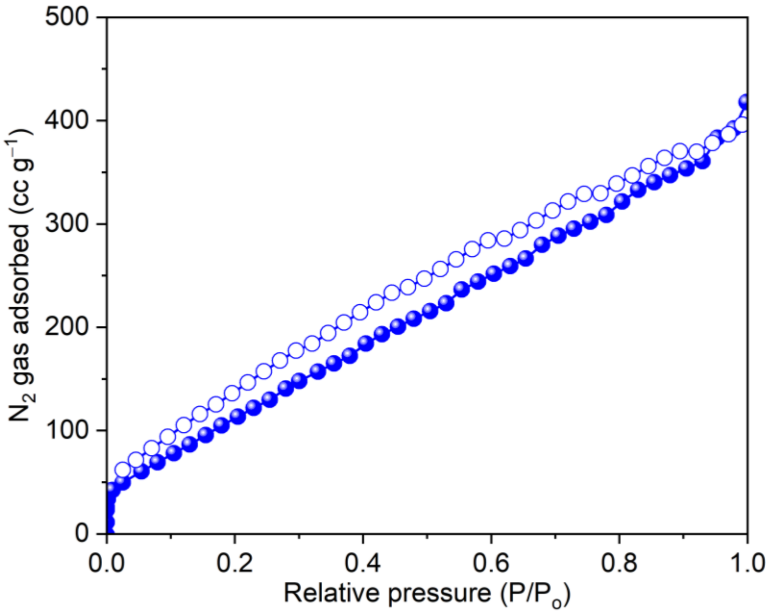

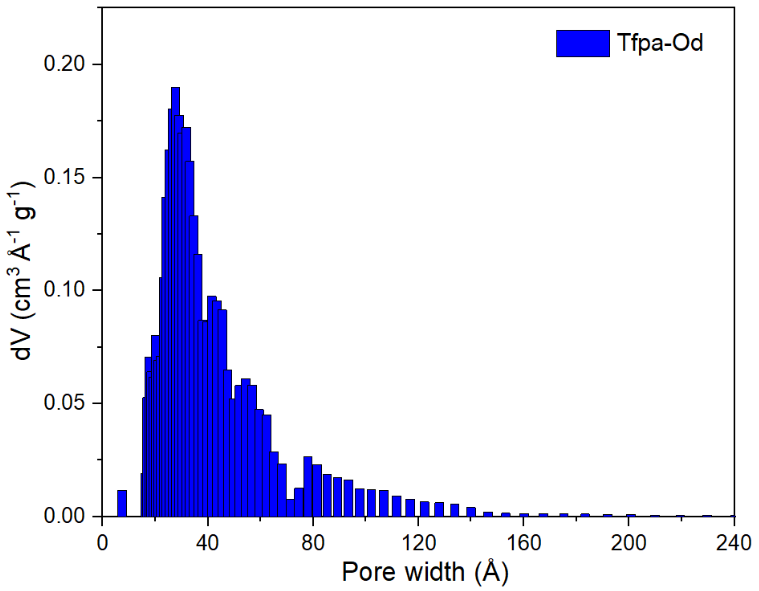

Figure S7. (top) N_2 gas adsorption isotherm of Tfpa-Od recorded at 77 K (surface area of 555 m^2 g^{-1}) and (bottom) Pore size distribution curve of Tfpa-Od calculated from NL-DFT method.

S3.5. Additional characterizations

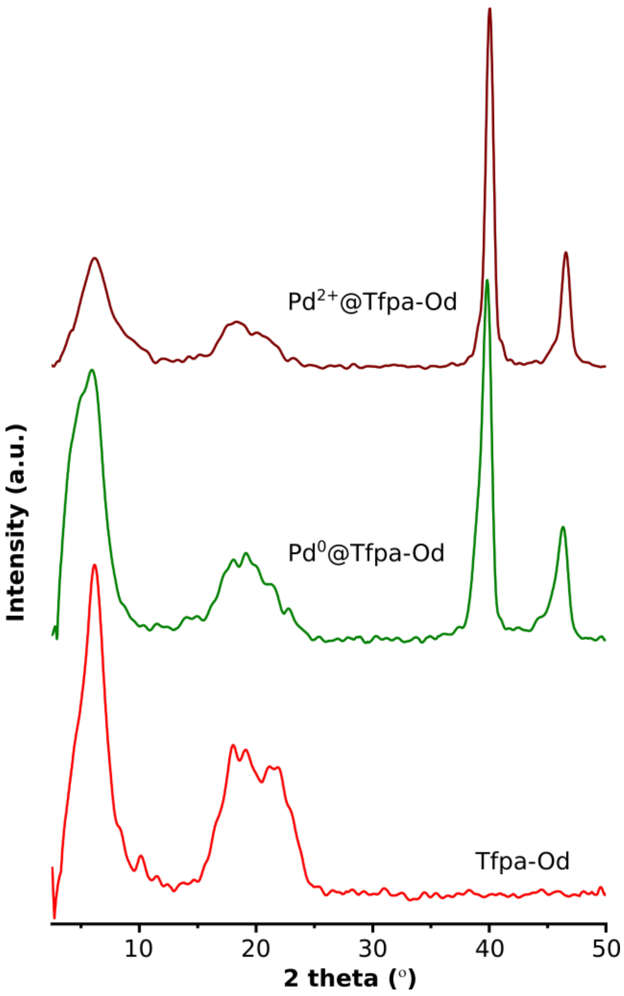

Figure S8. PXRD of **Pd^{2+}@Tfpa-Od** (brown)**, Pd-NP@Tfpa-Od** (green) and **Tfpa-Od** (red) after regeneration (cycle 4).

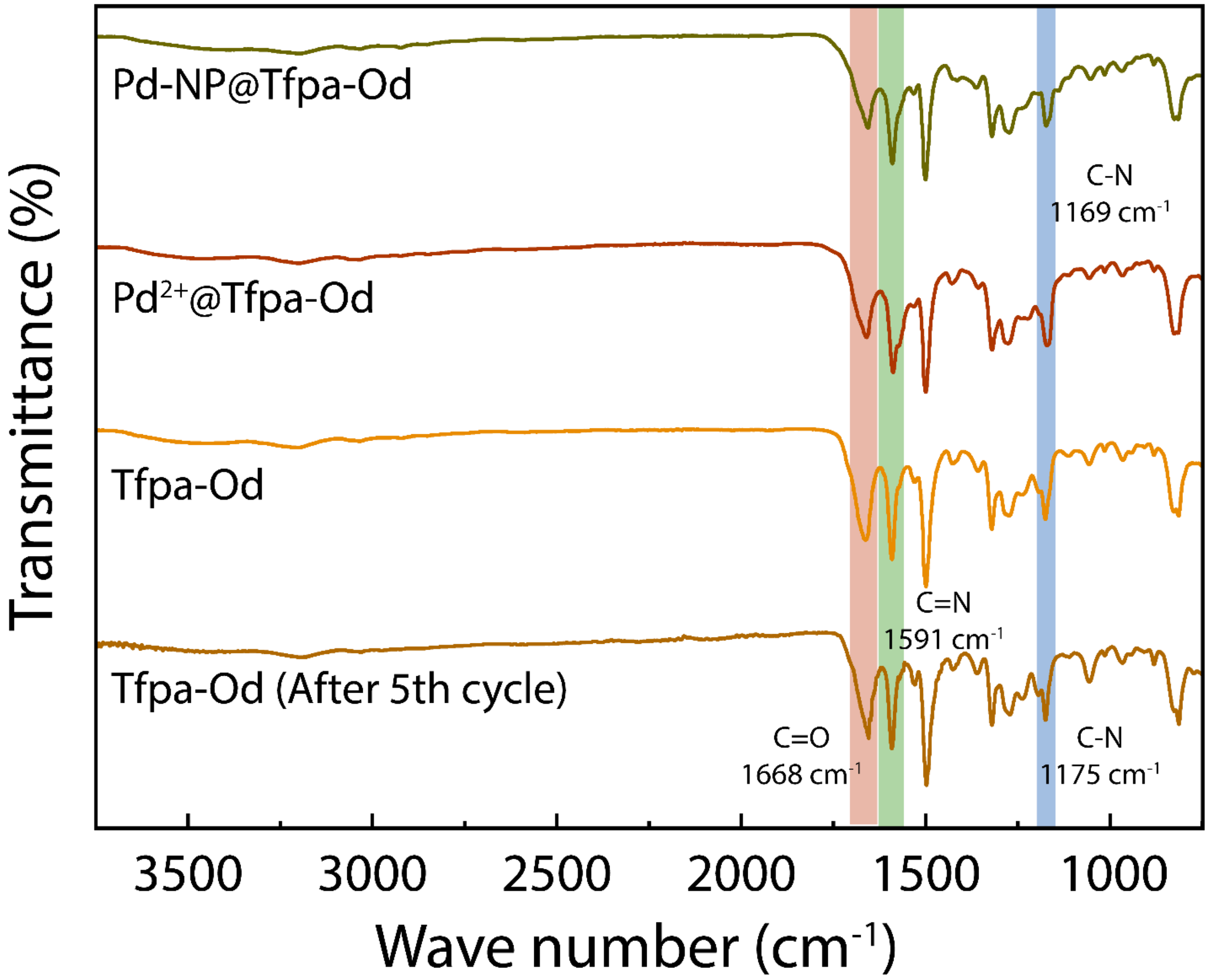

Figure S9. FT-IR spectra of **Tfpa-Od**, **Pd^{2+}@Tfpa-Od**, **Pd-NP@Tfpa-Od** and **Tfpa-Od** after desorption in the 5th cycle.

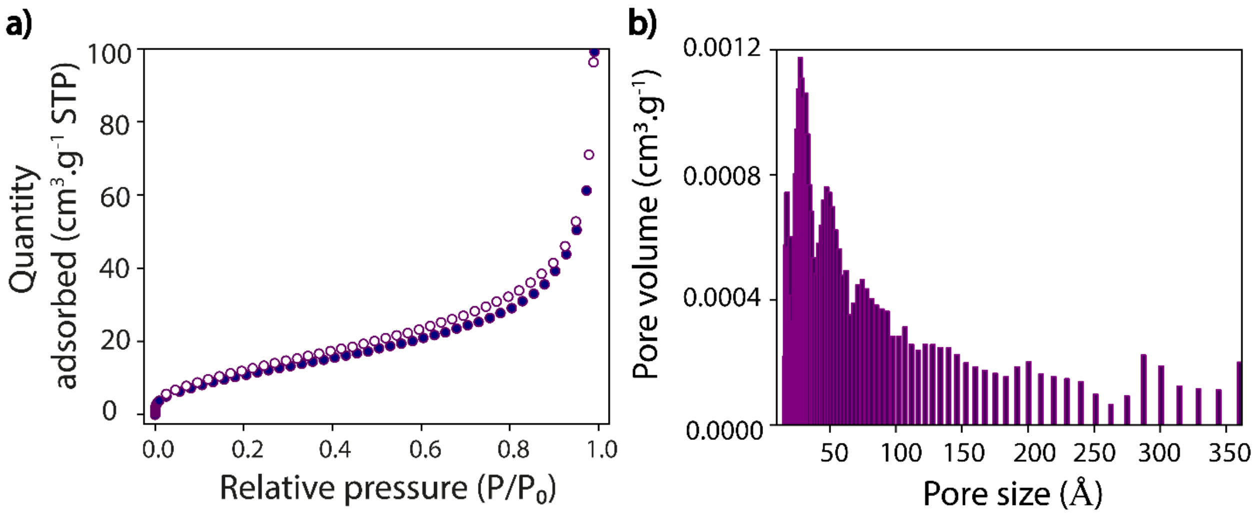

Figure S10. a) N_2 adsorption isotherm for **Pd^{2+}@Tfpa-Od**, and b) NLDFT pore size distribution. (BET surface area: 45.35 m^2 g^{-1})

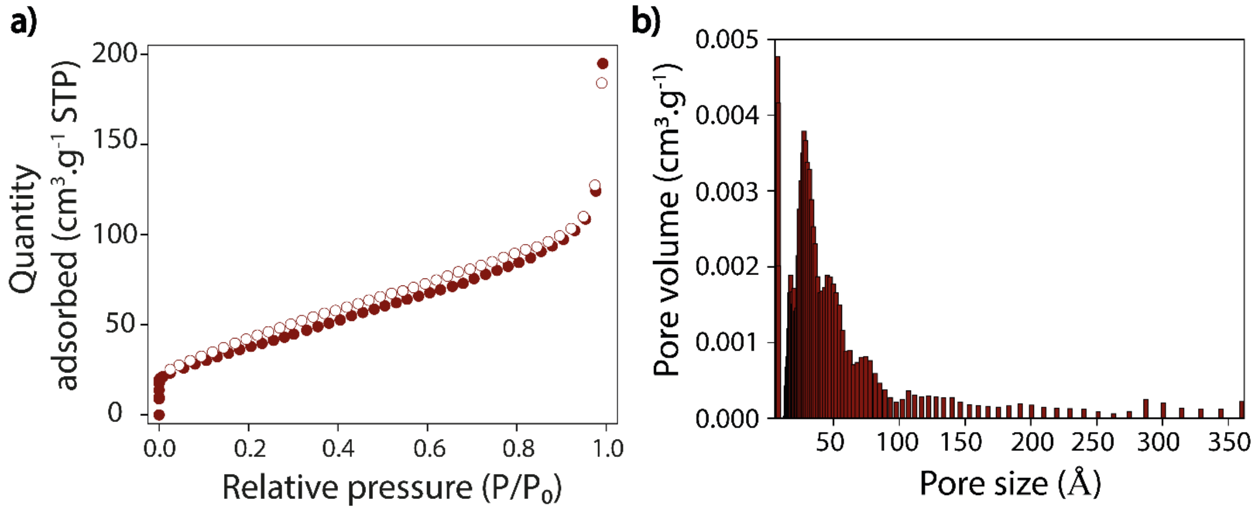

Figure S11. a) N_2 adsorption isotherm for Pd-NP@Tfpa-Od, and b) NLDFT pore size distribution. (BET surface area: 144.39 m^2 g^{-1})

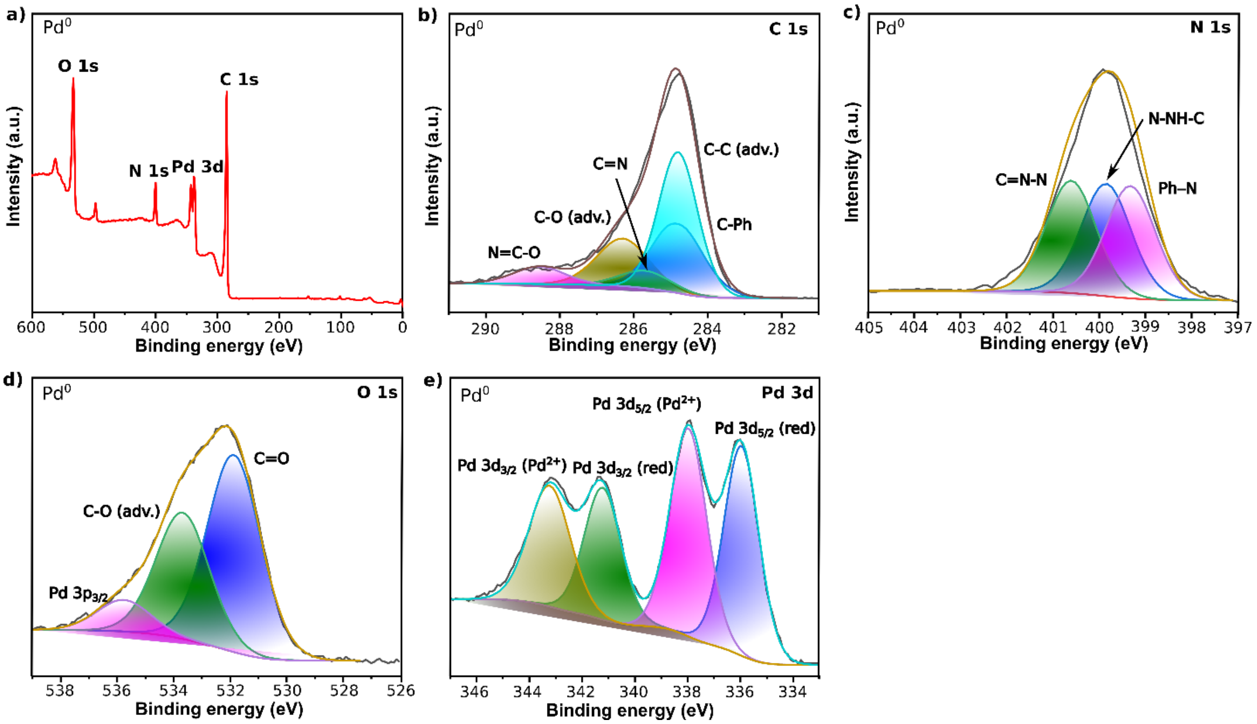

Figure S12. XPS spectra of **Pd-NP@Tfpa-Od.** a) Survey spectra, deconvoluted b) C 1s, c) N 1s, d) O 1s and e) Pd 3d spectra.

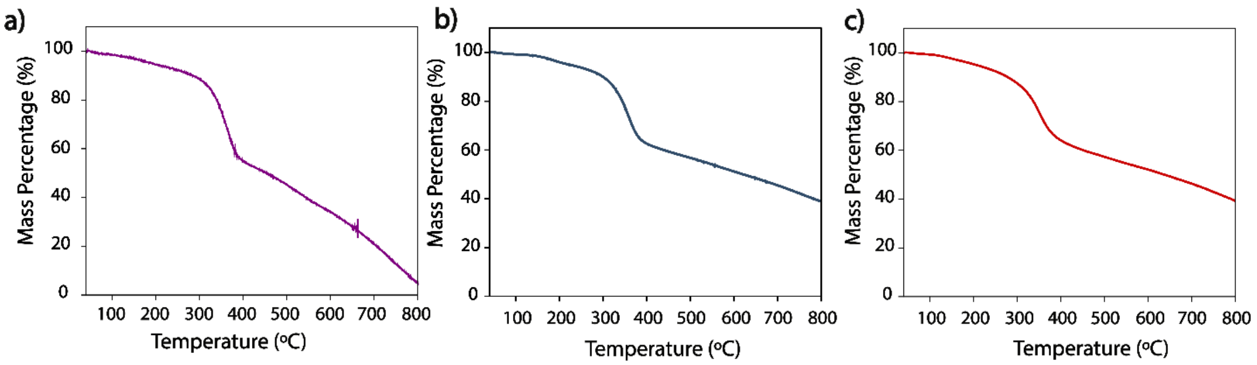

Figure S13. TGA plots of a) **Tfpa-Od**, b) **Pd^{2+}@Tfpa-Od** and c) **Pd-NP@Tfpa-Od**.

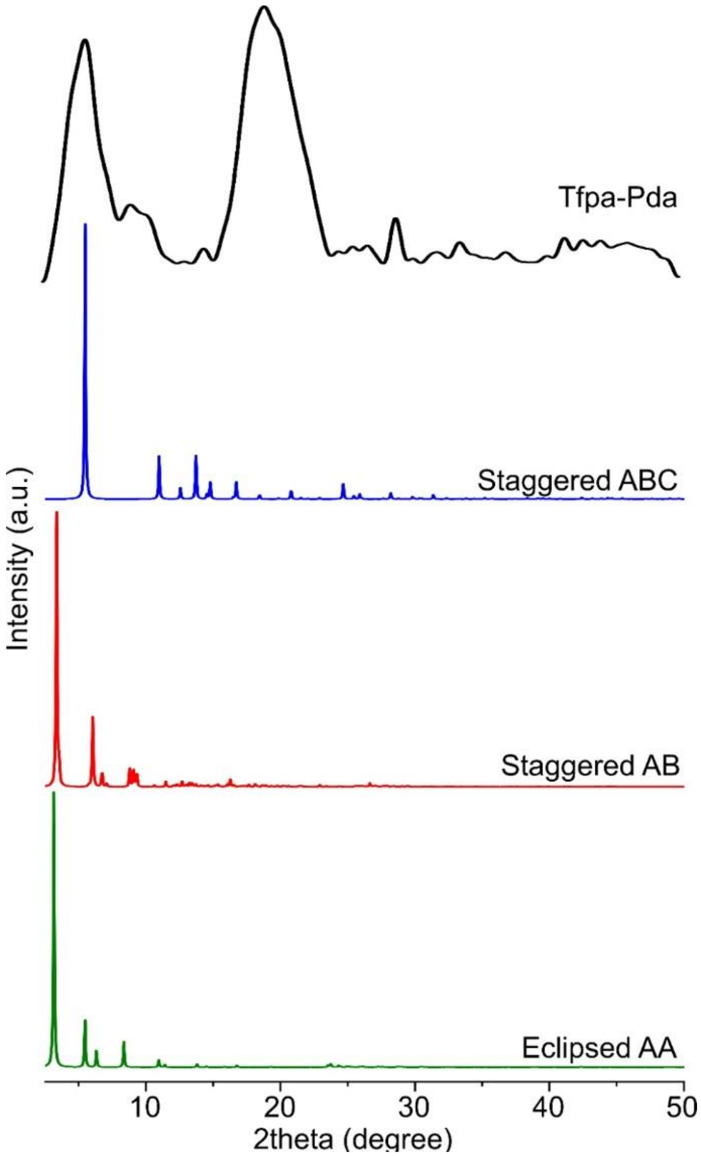

Figure S14. Comparison of experimental PXRD pattern with simulated ones found in agreement with ABC conformation model for Tfpa-Pda COF.

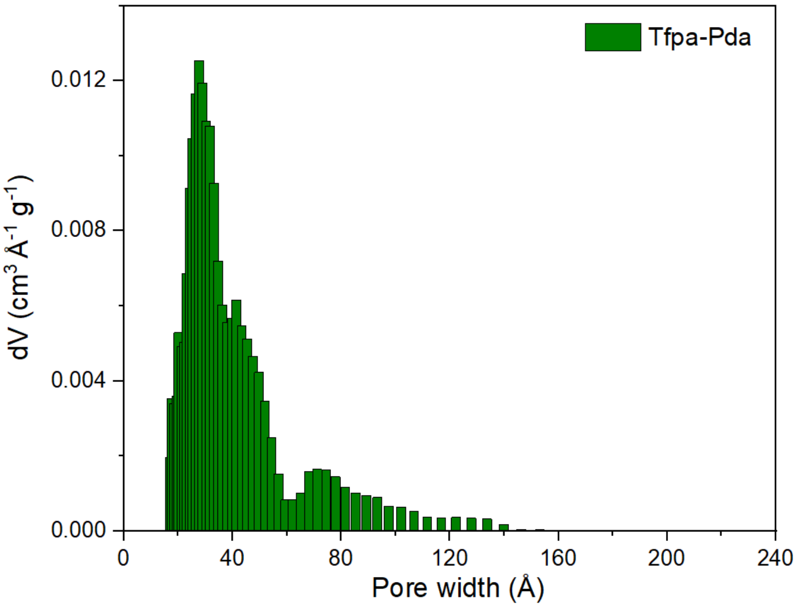

Figure S15. Pore size distribution curve of Tfpa-Pda calculated from NL-DFT method.

Section S4. Adsorption Studies

S4.1. Additional experimental and calculation details

Pd^{2+} adsorption experiments. 500 ppm Pd stock solution was prepared by dissolving $PdCl_2$ in distilled water using 200 mL standard flask. Lower concentrations (300, 200, 100, 50, 10, 5 ppm, etc.) were prepared by diluting the stock solution.

Equations for adsorption kinetics and isotherms

Adsorption kinetics. The obtained results were fitted with pseudo-second order equation (Equation 1).

$$t/q_t = (k_2\, q_e^{2})^{-1} + t/q_e \qquad (1)$$

Where, t is the time (min) and k_2 is the pseudo second order rate constant (g mg^{-1} min^{-1}). q_e and qt are the adsorption capacity (mg g^{-1}) at equilibrium and time t, respectively. q_t and q_e can be calculated as follows,

$$q_t = (C_0\text{-}C_t/M) \times V \qquad (2)$$

$$q_e = (C_0\text{-}C_e/M) \times V \qquad (3)$$

Uptake efficiency = R% = (C_0 -C_t/C_0) × 100 (4)

Where C_0 and C_t are the concentration in ppm at initial and time t, M is the mass of adsorbent in g, and V is the volume of solution in liter. The distribution coefficient (K_d) was calculated by multiplying q_e with $1/C_e$

Adsorption isotherm. The adsorption isotherm of the adsorbent towards a target adsorbate provides the mode/behavior of adsorption, maximum adsorption capacity, etc. The assumptions in Langmuir adsorption isotherm are that all the adsorption sites over the adsorbent surface are equivalent, there is no interaction between the adjacent adsorbate molecules, and each adsorption site can only occupy one adsorbate. Hence, it considers only monolayer chemical adsorption over the uniform adsorbent surface.

The Langmuir adsorption isotherm model can be expressed as follows:

$$C_e/q_e = (q_m \times K_a)^{-1} + C_e/q_e \qquad (5)$$

S4.2. Additional adsorption results

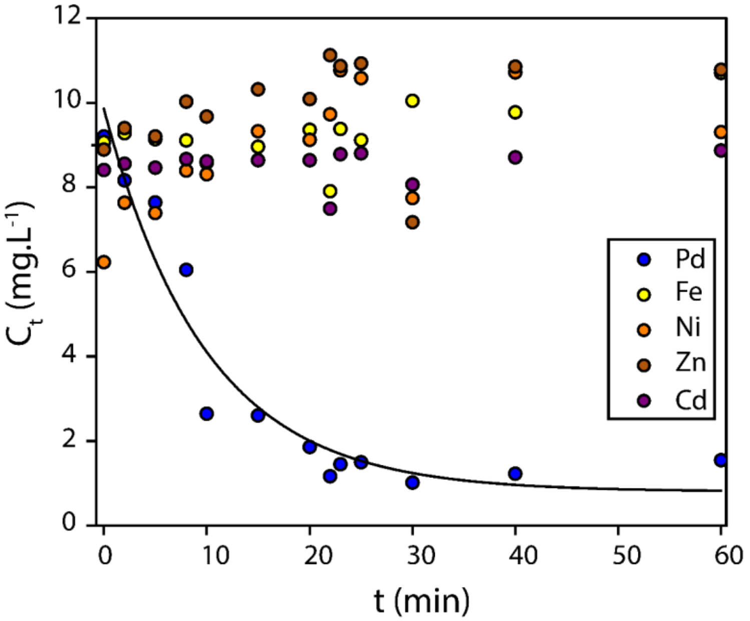

Figure S16. Kinetics of selectivity study for **Pd^{2+}@Tfpa-Od** along with other competing metal ions.

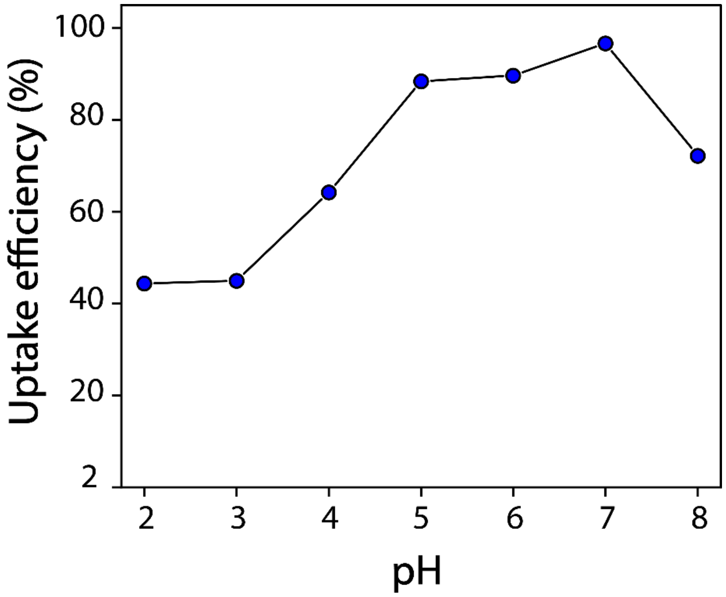

Figure S17. Effect of pH in the adsorption of Pd over **Tfpa-Od** in terms of uptake efficiency.

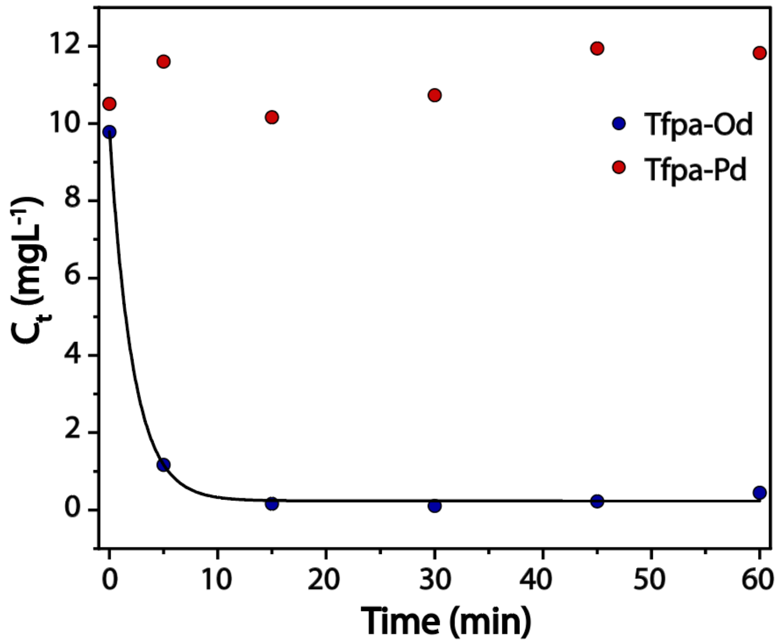

Figure S18. Adsorption performance comparison of **Tfpa-Pda** control imine COF for Pd uptake with **Tfpa-Od**.

Section S5. Catalytic Study

S5.1. NMR data and spectra

4-Nitro-1,1'-biphenyl. (yellow crystalline solid), NMR yield: >99%; isolated yield: 96% (95.8 mg); ^{1}H NMR (500 MHz, $CDCl_3$): δ 8.30 (d, J = 8.8 Hz, 2H), 7.75 (d, J = 8.8 Hz, 2H), 7.63 (d, J = 7.8 Hz, 2H), 7.50 (dd, J = 7.1 Hz and 7.6 Hz, 2H), 7.46 (d, J = 7.3 Hz, 1H). $^{13}C\{^1H\}$ NMR (125 MHz, $CDCl_3$): δ 147.7, 147.2, 138.9, 129.3, 129.0, 127.9, 127.5, 124.2.

[1,1'-Biphenyl]-4-ol. (white crystalline solid), NMR yield: 91%; isolated yield: 89% (76 mg); ^{1}H NMR (500 MHz, $CDCl_3$): δ 7.56 (d, J = 7.4 Hz, 2H), 7.50 (d, J = 8.3 Hz, 2H), 7.44 (t, J = 7.3 Hz, 2H), 7.33 (t, J = 7.2 Hz, 1H), 6.93 (d, J = 8.2 Hz, 2H), 5.14 (s, 1H). $^{13}C\{^1H\}$ NMR (125 MHz, $CDCl_3$): δ 155.2, 140.9, 134.1, 128.9, 128.5, 126.9, 115.8.

[1,1'-Biphenyl]-2-ol. (white solid), NMR yield: 85%; isolated yield: 83% (70.6 mg); ^{1}H NMR (400 MHz, $CDCl_3$): δ 7.51-7.48 (m, 4H), 7.44-7.39 (m, 1H), 7.30-7.25 (m, 2H), 7.03-6.99 (m, 2H), 5.27 (s, 1H). $^{13}C\{^1H\}$ NMR (100 MHz, $CDCl_3$): δ 152.5, 137.2, 130.4, 129.4, 129.3, 129.2, 128.2, 128.0, 120.9, 115.9.

4-Methoxy-1,1'-biphenyl. (white solid), NMR yield: 79%; isolated yield: 76% (70 mg); ^{1}H NMR (500 MHz, $CDCl_3$): δ 7.56-7.53 (m, 4H), 7.43 (t, J = 7.6 Hz, 2H), 7.32 (t, J = 7.4 Hz, 1H), 6.99 (d, J = 8.8 Hz, 2H), 3.86 (s, 3H). $^{13}C\{^1H\}$ NMR (125 MHz, $CDCl_3$): δ 159.3, 140.9, 133.9, 128.9, 128.3, 126.9, 126.8, 114.6, 55.5.

3-(Trifluoromethyl)-1,1'-biphenyl. (colourless oil), NMR yield: >99%; isolated yield: 95% (105.5 mg); ^{1}H NMR (400 MHz, $CDCl_3$): δ 7.86 (s, 1H), 7.79 (d, J = 7.6 Hz, 1H), 7.62-7.60 (m, 3H), 7.59 (t, J = 7.7 Hz, 1H), 7.51-7.47 (m, 2H), 7.43 (t, 7.3 Hz, 1H). $^{13}C\{^1H\}$ NMR (100 MHz, $CDCl_3$): δ 142.2, 139.9, 131.3 (q, J = 32 Hz), 130.6, 129.4, 129.1, 128.2, 127.3, 124.3 (q, J = 272 Hz), 124.1 (q, J = 3.5 Hz), 124.0 (q, J = 3.5 Hz).

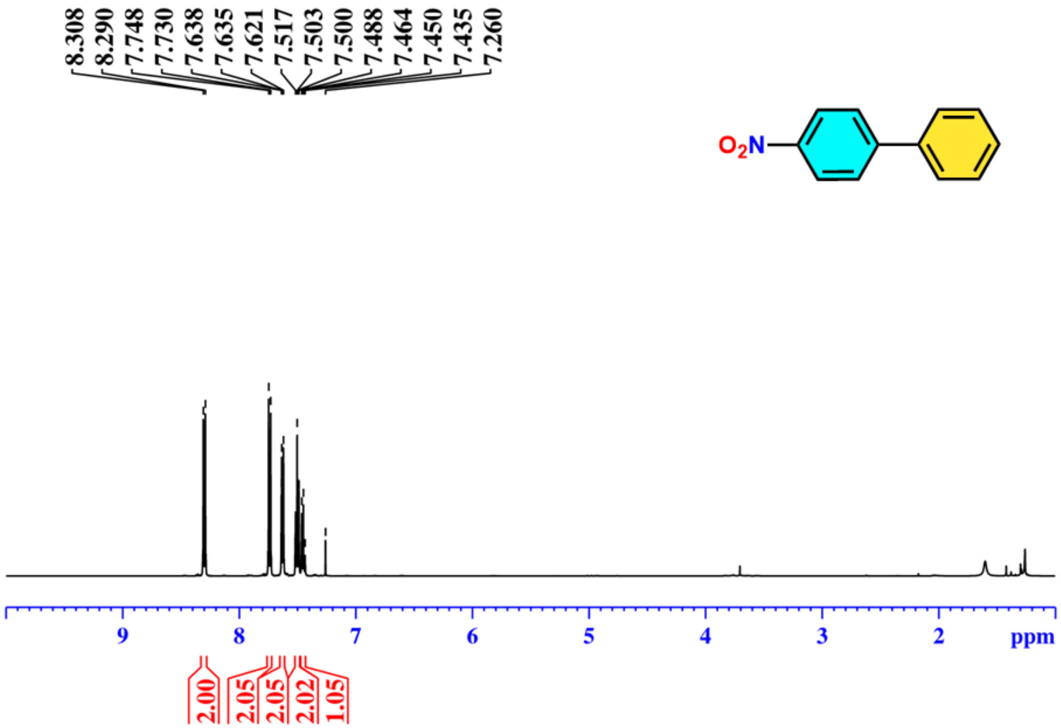

Figure S19. ^{1}H NMR spectrum of crude 4-nitro-1,1'-biphenyl recorded in $CDCl_3$.

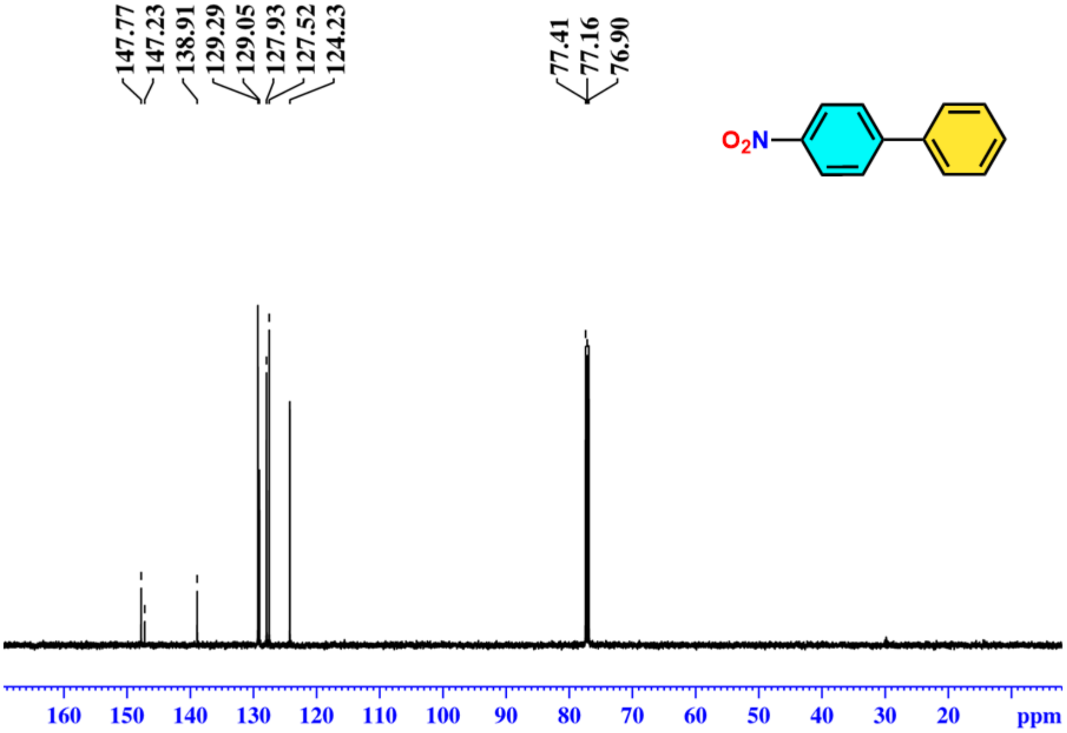

Figure S20. ^{13}C NMR spectrum of crude 4-nitro-1,1'-biphenyl recorded in $CDCl_3$.

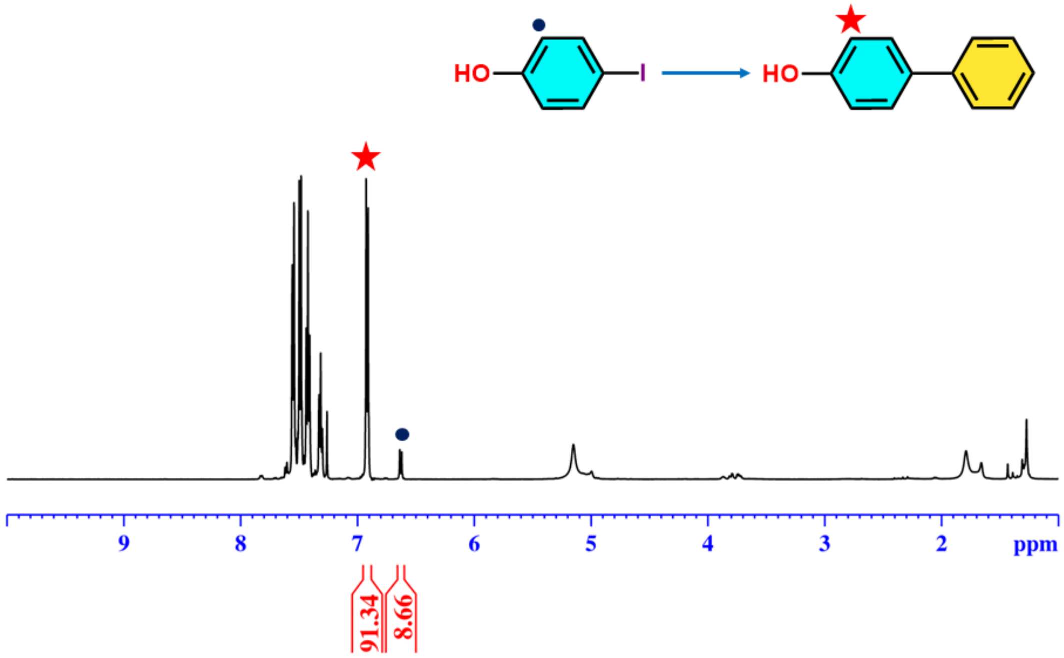

Figure S21. ^{1}H NMR spectrum of crude [1,1'-biphenyl]-4-ol recorded in $CDCl_3$.

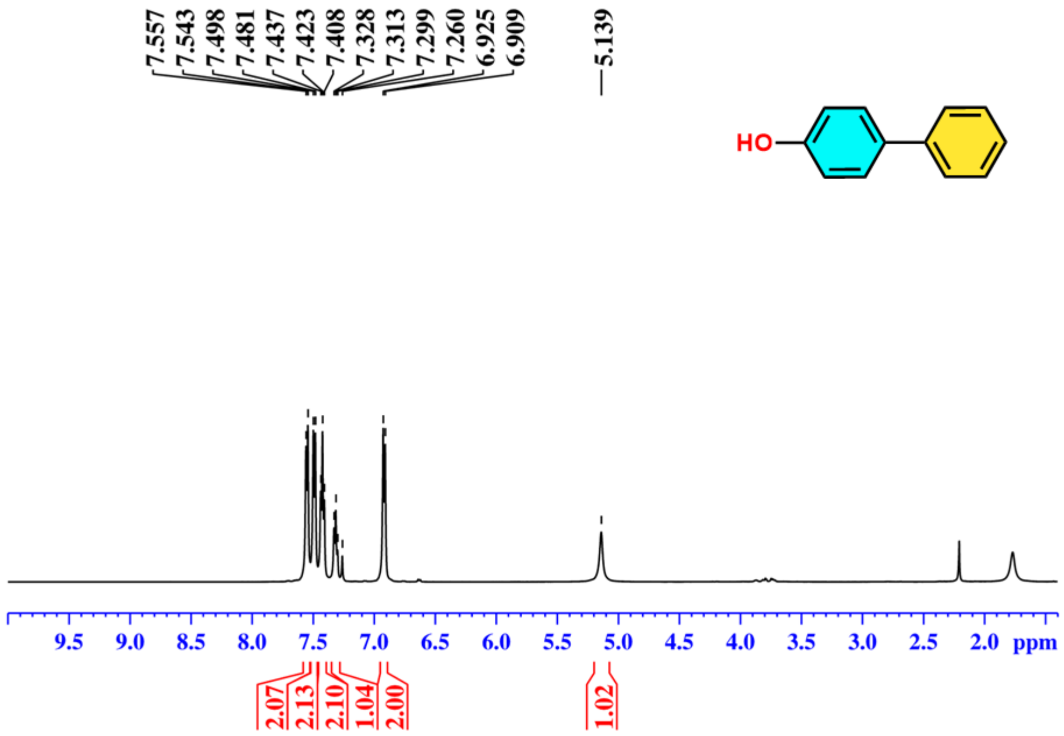

Figure S22. ^{1}H NMR spectrum of purified [1,1'-biphenyl]-4-ol recorded in $CDCl_3$.

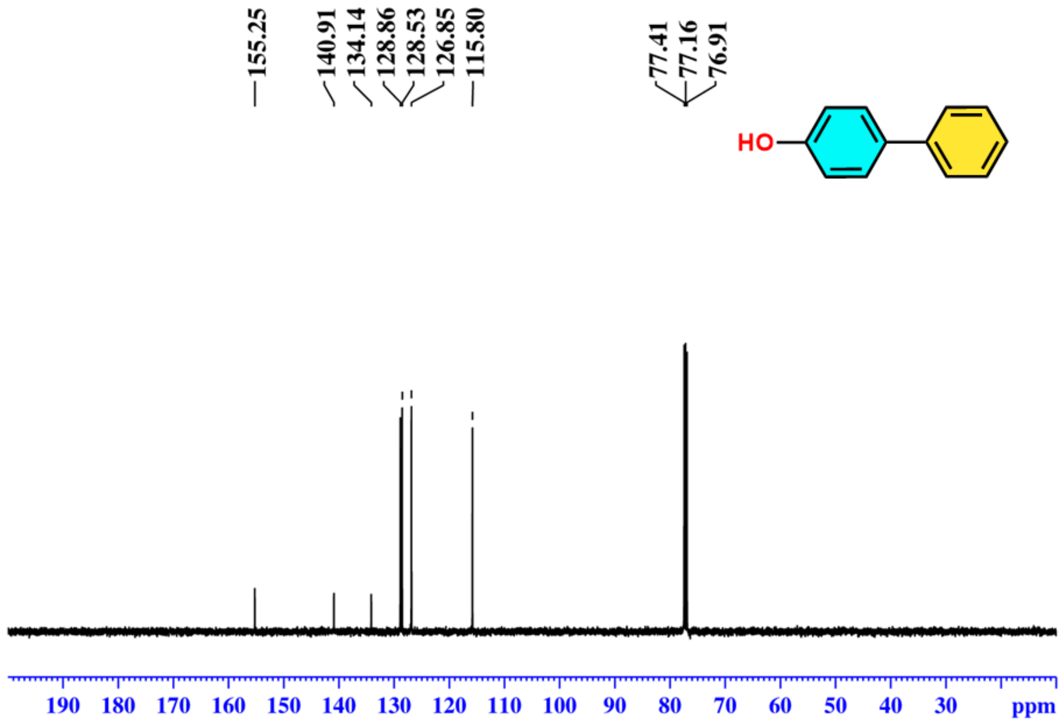

Figure S23. ^{13}C NMR spectrum of purified [1,1'-biphenyl]-4-ol recorded in $CDCl_3$.

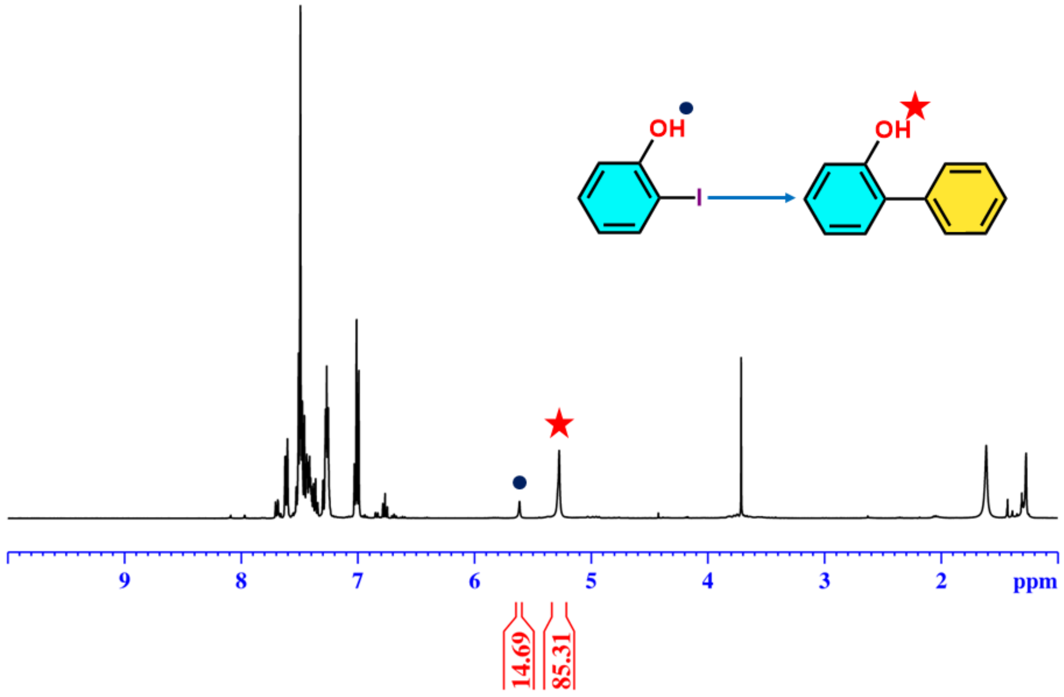

Figure S24. ^{1}H NMR spectrum of crude [1,1'-biphenyl]-2-ol recorded in $CDCl_3$.

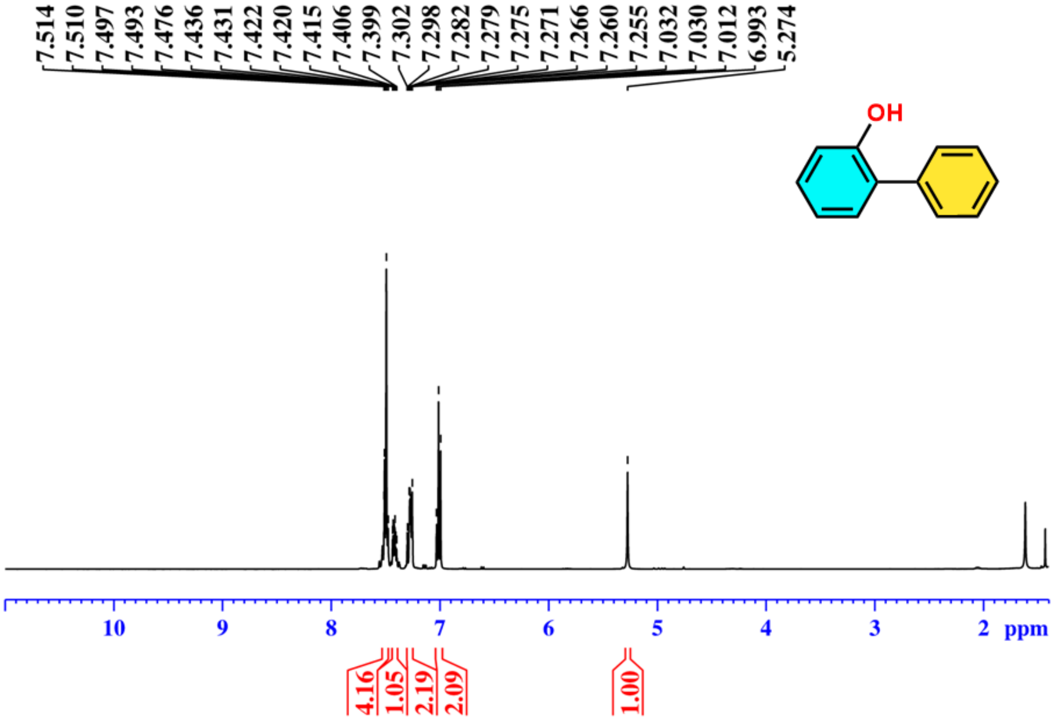

Figure S25. ^{1}H NMR spectrum of purified [1,1'-biphenyl]-2-ol recorded in $CDCl_3$.

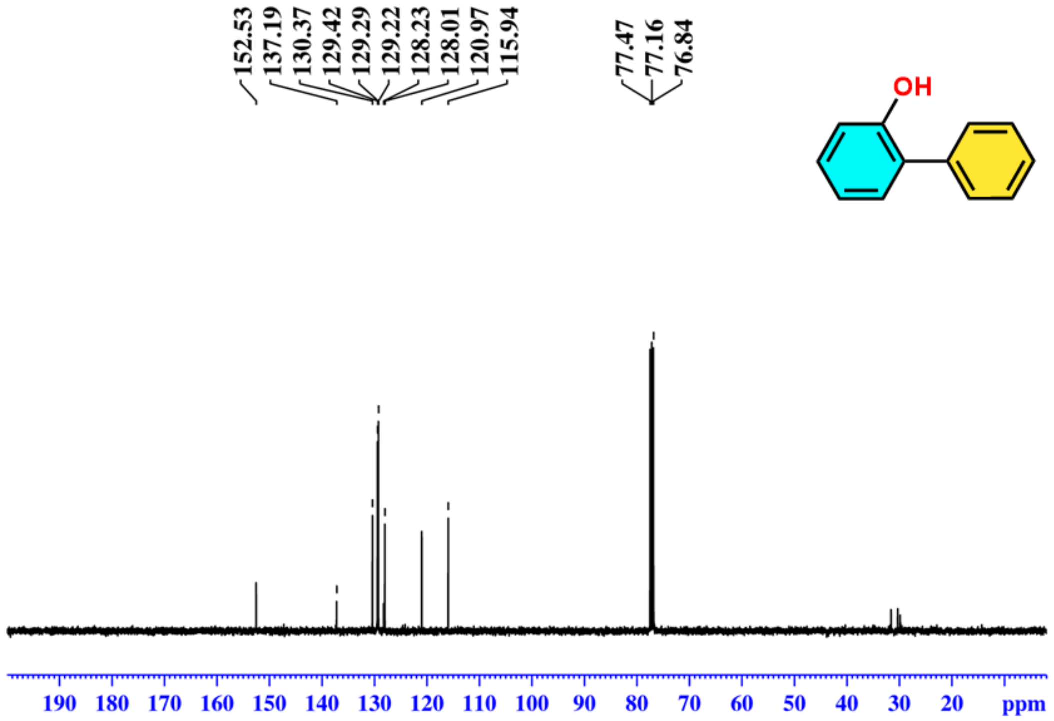

Figure S26. ^{13}C NMR spectrum of purified [1,1'-biphenyl]-2-ol recorded in $CDCl_3$.

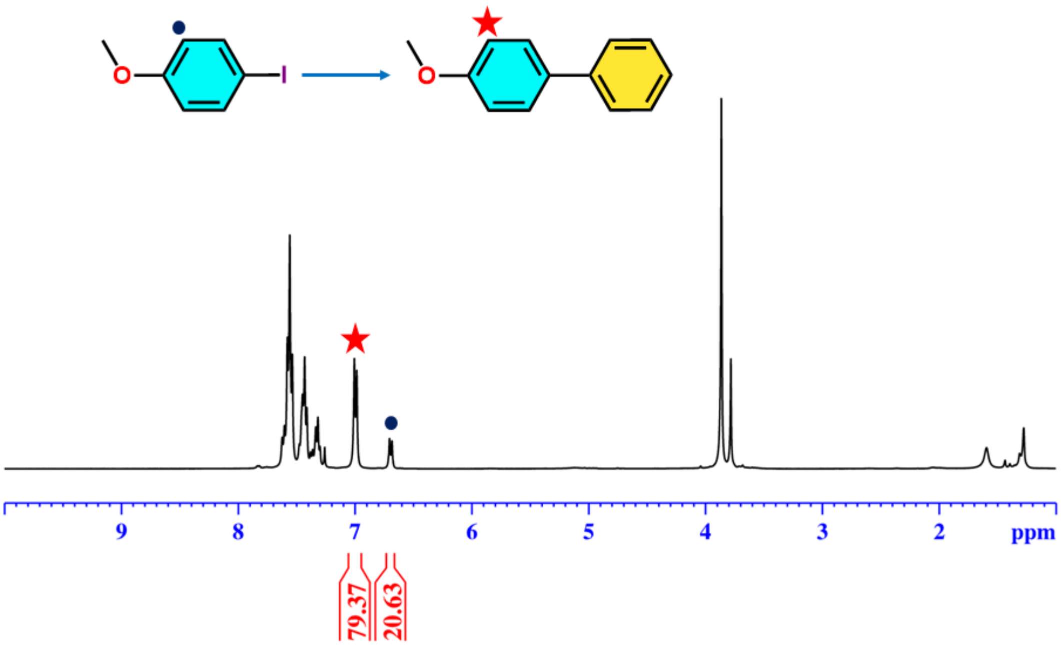

Figure S27. ^{1}H NMR spectrum of crude 4-methoxy-1,1'-biphenyl recorded in $CDCl_3$.

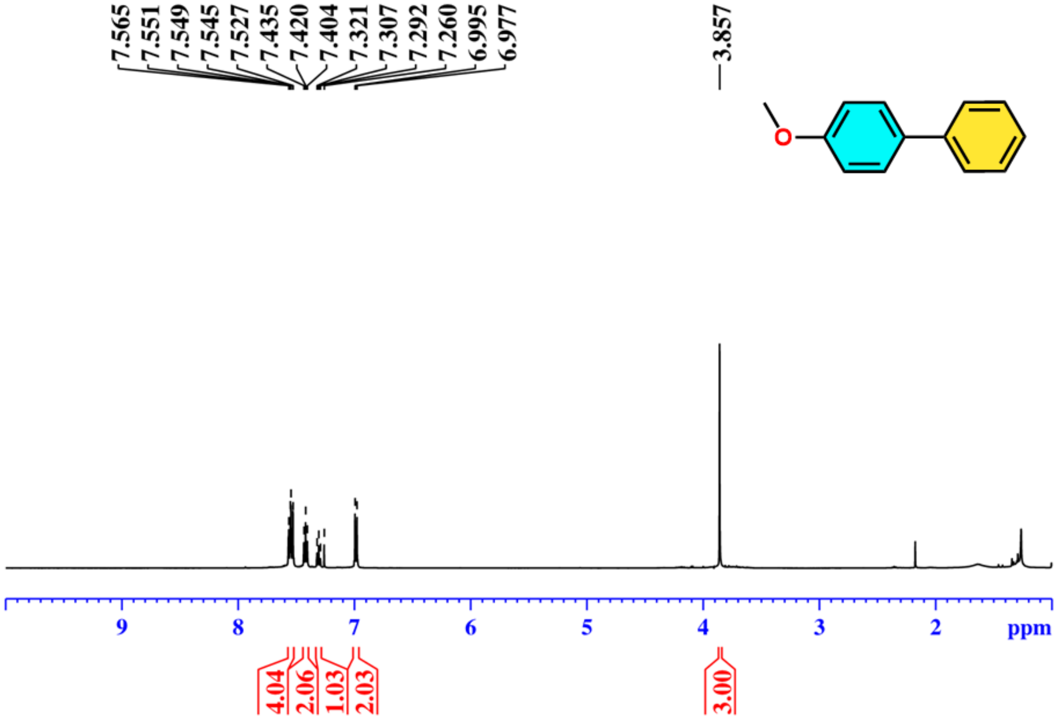

Figure S28. ^{1}H NMR spectrum of purified 4-methoxy-1,1'-biphenyl recorded in $CDCl_3$.

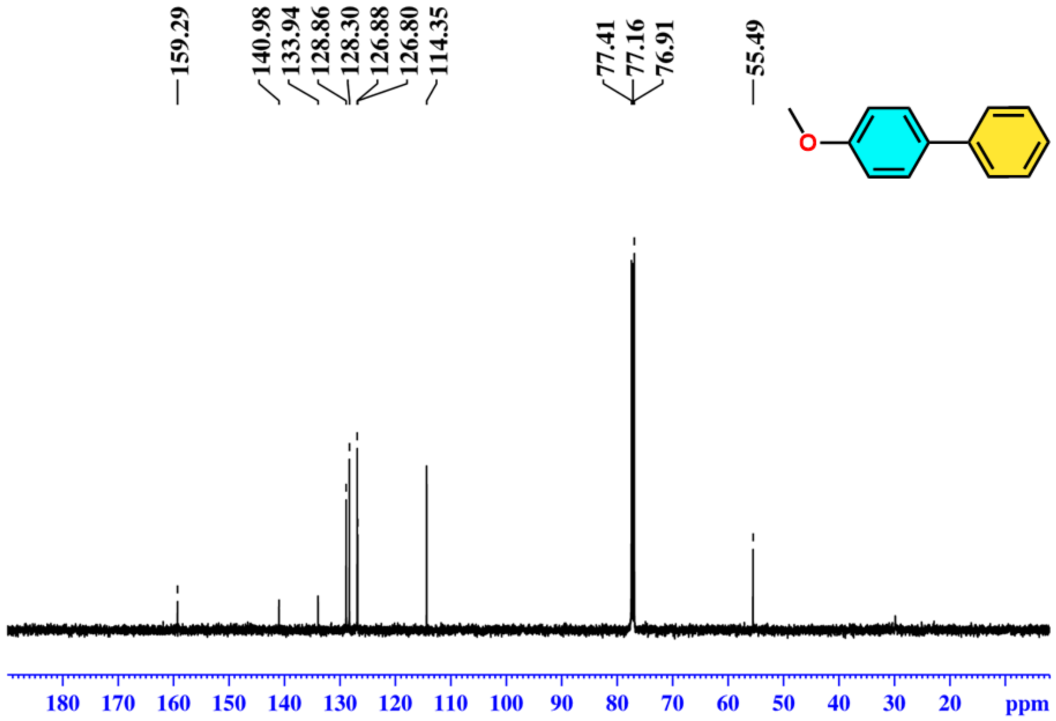

Figure S29. ^{13}C NMR spectrum of purified 4-methoxy-1,1'-biphenyl recorded in $CDCl_3$.

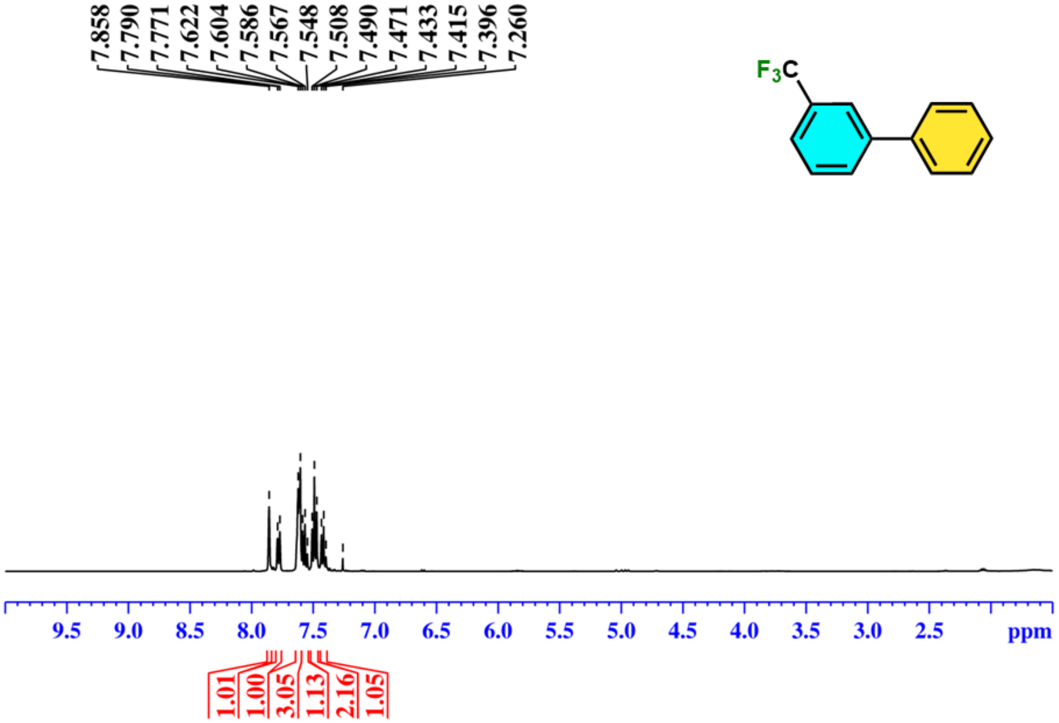

Figure S30. ^{1}H NMR spectrum of crude 3-(trifluoromethyl)-1,1'-biphenyl recorded in $CDCl_3$.

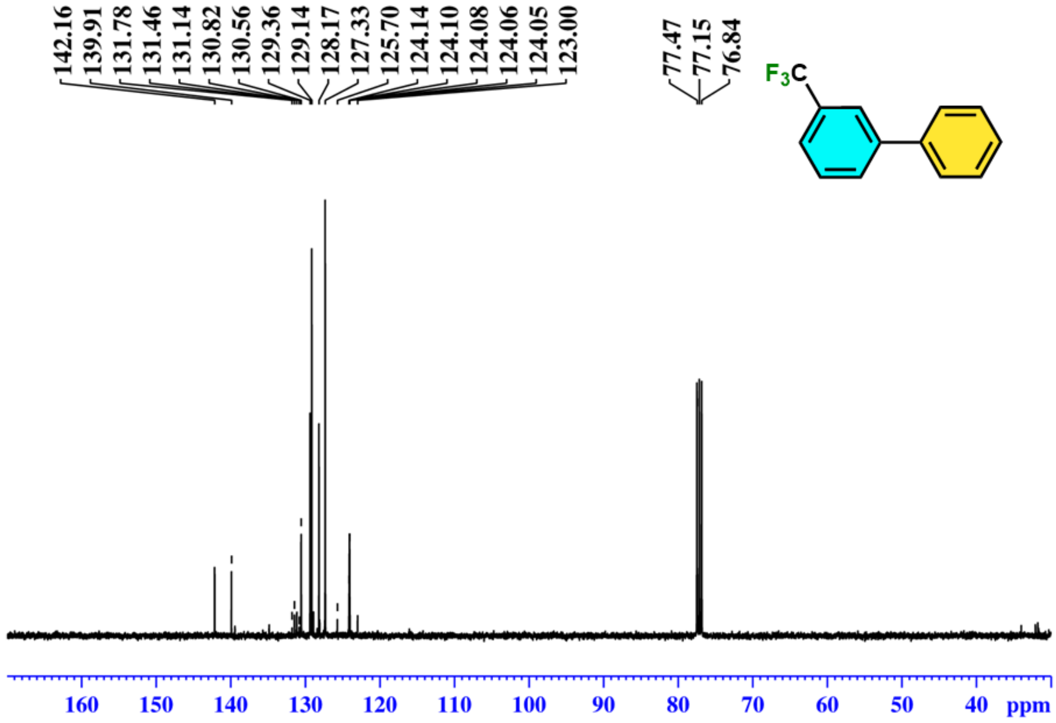

Figure S31. ^{13}C NMR spectrum of crude 3-(trifluoromethyl)-1,1'-biphenyl recorded in $CDCl_3$.

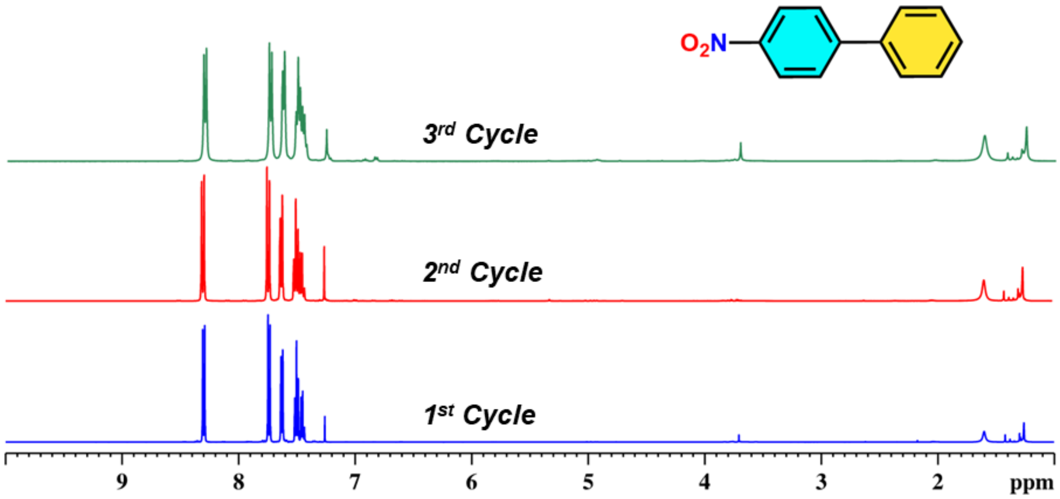

Figure S32. Stacked ^{1}H NMR spectra of crude 4-Nitro-1,1'-biphenyl after each cycle recorded in $CDCl_3$.

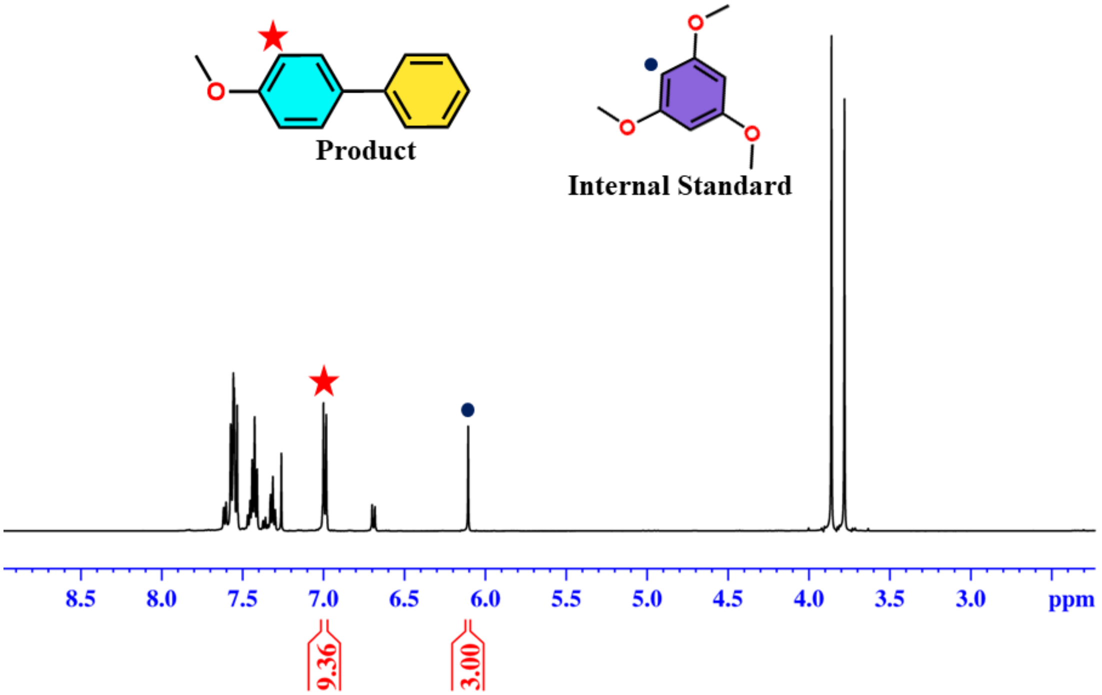

Figure S33. ^{1}H NMR of part of the total crude mixture (12 mg) in the presence of internal standard (0.01 mmol) in $CDCl_3$.

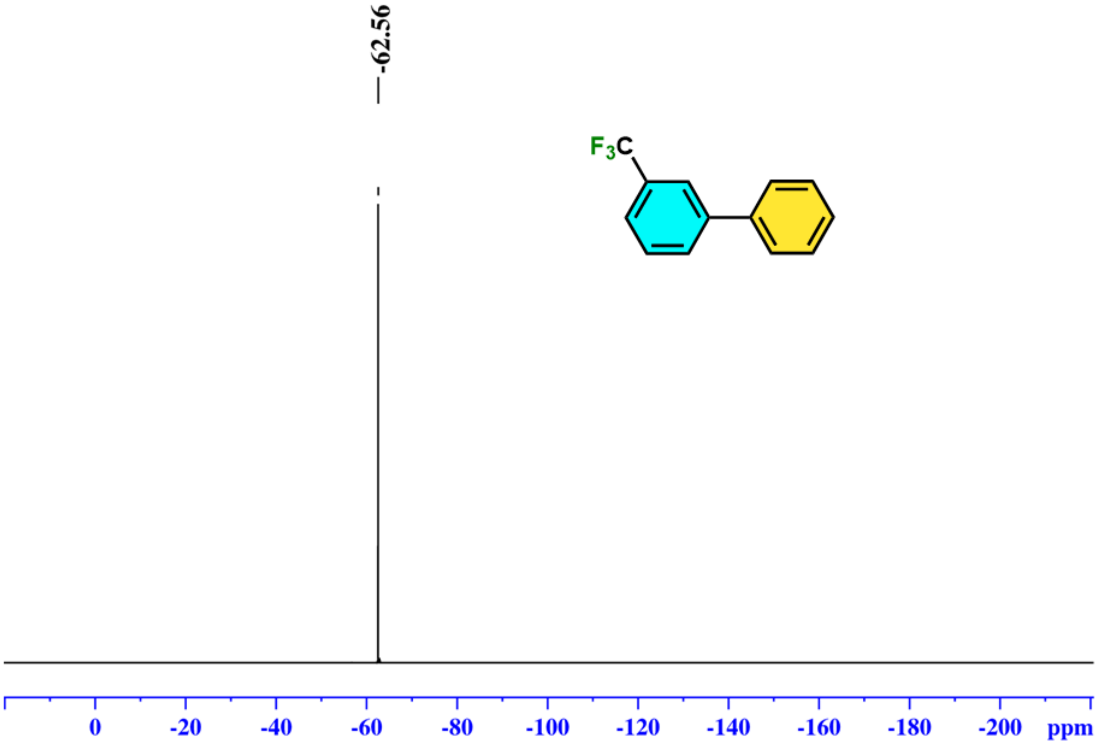

Figure S34. ^{19}F NMR of 3-(trifluoromethyl)-1,1'-biphenyl recorded in $CDCl_3$.

S5.2. Additional results in catalytic study

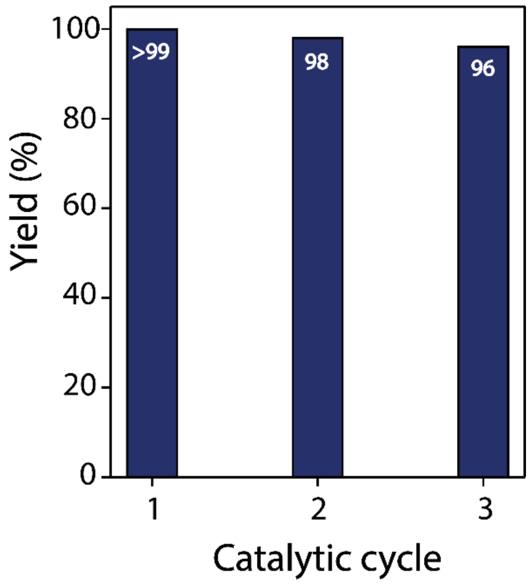

Figure S35. Catalytic recyclability of **Pd-NP@Tfpa-Od** for Suzuki-Miyaura coupling reaction.

Table S4. Mass of the recovered COF compound for each cycle in the catalytic recyclability tests during the Suzuki-Miyaura couplings

Cycle	Recovered mass of Pd-NP@Tfpa-Od
**First cycle**	2 mg (starting catalyst amount)
**Second cycle**	1.9 mg (recovered from the first cycle)
**Third cycle**	1.7 mg (recovered from the second cycle)

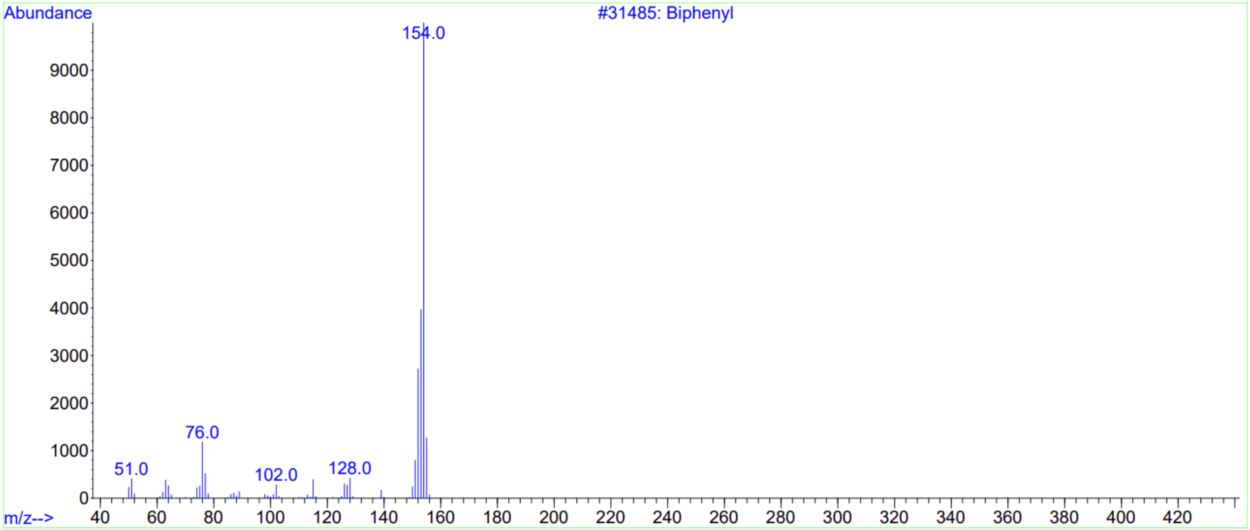

Figure S36. Mass spectra of Biphenyl obtained in the reaction between iodobenzene and phenylboronic acid. (For entry 1 in Table 1)

Section S6. Literature Tables

Table S5. Comparison of performance of **Pd@Tfpa-Od** for Suzuki-Miyaura coupling reaction with respect to reported COF-based catalysts.

Sl. No.	Pd loaded COFs	Solvent	Temperature (°C)	Yield	Stability cycle	Ref.
**1**	Pd/COF-LZU1	p-Xylene	150	97	5	6
**2**	(Pd/C)@TpPa	EtOH/H2O	30	87	5	7
**3**	Pd0/TAT–TFP	DMF	120	83	5	8
**4**	Pd/TATAE	Water	RT	98	4	9
**5**	Pd@COF-TM	Water	RT	99	9	10
**6**	Pd@OC-MA	Water	RT	99	4	11
**7**	PdNPs@Thio-COF	DMF/H2O	50	85	5	12
**8**	Pd/COF-SMC2	EtOH	80	96	4	13
**9**	TAPB–BTCA	THF	80	51	-	14
**10**	Pd@TPM-3D-COF-Bpy	DMF/H2O	70	98	5	15
**11**	Pd(II)@SP-3D-COF-Bpy	p-Xylene	70	98	5	16
**12**	PdNPs@Phos-COF-1a	DMF/H2O	50	>99	5	17
**13**	Pd-NP@Tfpa-Od	1,4-Dioxane	90	>99	3	This work

Table S6. Comparison of the present work with the published COF literature for recovery of precious metals from wastewater.

COF-based Materials	Target precious metals	Equilibrium time for adsorption	Adsorption capacity (mg g^{-1})	Distribution coefficient, ($\times 10^5$ mL g^{-1})	Adsorption mechanism	Ref.
**TpODH**	Cu(II)	200 min	324	-	Electrostatic interaction	18
**POFct-1**	Cu(II)	1500 min	135.60	1.01	Coordination with O and N	19
**QG- scaffolded COFs**	Cu(II)	40 s		-	Coordination with N	20
**TpPa-NH_2@EDTA**	Cu(II), Ag(I), Pd(II)	5 min	50	-	Coordination with EDTA	21
**TTB-COF**	Au(III)	1 min	560	-	Coordination with thioether moieties	22
**COF-316-DM**	Pd(II)	60 min	147.7	7.987×10^{-7}	Interaction of metal ion with thioamide groups	23
**XB-COF**	Pd(II)	140 min	120	-	Complexation of metal with allyl sidechains	24
**Tp-DG_{Cl}**	Pd(II)	300 min	342.1	0.8012	Electrostatic interaction	25
**Tfpa-Od COF**	Pd(II)	25 min	372.59	36.2	Coordination with N and O	This work

Table S7. Comparison of Tfpa-Od regarding Pd adsorption with respect to other reported adsorbents.

Adsorbent	Binding Motif	Adsorption Capacity (mg g^{-1})	Distribution Co-efficient (mL g^{-1})	Ref.
**Activated carbon**	Pd-pi interaction	35.71	-	26
**Fe_3O_4-MWCNTs**	Pd-pi interaction	112	-	27
**Graphene oxide**	Pd-O	80.7	-	28
**UiO-66**	Zr-oxo cluster	120	-	29
**MOF-808**	Zr-oxo cluster	163.9	-	30
**UiO-66-NH2**	Zr-oxo cluster	167	-	31
**MIL-101-NH2**	Protonated amine	119.5	1.8×10^4	32
**AHPP-MOF**	N and O atoms	283.5	-	33
**Crown ether functionalized Silica**	O in crown ether	83.3	0.0173	34
**SiNC-CTAB**	CTAB units	124.5	1.86×10^5	35
**PMA-SNP**	-NH and -OH groups	53.61	9×10^4	36
**Biopolymer alginate-MIAC**	N and O atoms	316.92	2.15×10^4	37
**CuS-NPs**	S atoms	222.2	2.24×10^6	38
**CITCF-500**	Trithiocyanurate ring (Pd-S)	909.1	5.6×10^5	39
**Tfpa-Od**	Hydrazone linkage	372.59	3.62×10^6	This work

Section S7. References

(1) Gándara, F.; Bennet, T.D. Crystallography of metal-organic frameworks, *International Union of Crystallography (IUCrJ)*, **2014**, *28*, 563-70.

(2) Gropp, C.; Canossa, S.; Wuttke, S.; Gándara, F.; Li, Q.; Gagliardi, L.; Yaghi, O. M. Standard practices of reticular chemistry, *ACS Central Science*, **2020**, *6*, 1255-1273.

(3) Côté, A. P.; Benin, A. I.; Ockwig, N. W.; O'keeffe, M.; Matzger, A. J.; Yaghi, O. M. Porous, Crystalline, Covalent Organic Frameworks, *Science*, **2005**, *310*, 1166.

(4) Kandambeth, S.; Mallick, A.; Lukose, B.; Mane, M. V.; Heine, T.; Banerjee, R., *J. Am. Chem. Soc.*, **2012**, *134*, 19524.

(5) Huang, N.; Wang, P.; Jiang, D. Covalent organic frameworks: a materials platform for structural and functional designs, *Nat. Rev. Mater.*, **2016**, 16068.

(6) Ding, S.; Gao, J.; Wang, Q.; Zhang, Y.; Song, W.; Su, C.; Wang, W. Construction of covalent organic framework for catalysis: Pd/COF-LZU1 in Suzuki–Miyaura coupling reaction. *J. Am. Chem. Soc.* **2011**, *133*, 19816-19822.

(7) Li, Y.; Pei, B.; Chen, J.; Bing, S.; Hou, L.; Sun, Q.; Xu, G.; Yao, Z.; Zhang, L. Hollow nanosphere construction of covalent organic frameworks for catalysis:(Pd/C)@ TpPa COFs in Suzuki coupling reaction. *J. Colloid Interface Sci.* **2021**, *591*, 273-280.

(8) Kaleeswaran, D.; Antony, R.; Sharma, A.; Malani, A.; Murugavel, R. Catalysis and CO2 Capture by Palladium- Incorporated Covalent Organic Frameworks. *ChemPlusChem* **2017**, *82*, 1253-1265.

(9) Sadhasivam, V.; Balasaravanan, R.; Chithiraikumar, C.; Siva, A. Incorporating Pd (OAc) 2 on Imine Functionalized Microporous Covalent Organic Frameworks: A Stable and Efficient Heterogeneous Catalyst for Suzuki- Miyaura Coupling in Aqueous Medium. *ChemistrySelect* **2017**, *2*, 1063-1070.

(10) Wu, S.; Ding, N.; Jiang, P.; Wu, L.; Feng, Q.; Zhao, L.; Wang, Y.; Su, Q.; Zhang, H.; Yang, Q. A two-dimensional amide-linked covalent organic framework anchored Pd catalyst for Suzuki-Miyaura coupling reaction in the aqueous phase at room temperature. *Tetrahedron Lett.* **2020**, *61*, 152656.

(11) Dong, Z.; Pan, H.; Gao, P.; Xiao, Y.; Fan, L.; Chen, J.; Wang, W. Palladium Immobilized on a Polyimide Covalent Organic Framework: An Efficient and Recyclable

Heterogeneous Catalyst for the Suzuki–Miyaura Coupling Reaction and Nitroarene Reduction in Water. *Catalysis Letters* **2022**, *152*, 299-306.

(12) Lu, S.; Hu, Y.; Wan, S.; McCaffrey, R.; Jin, Y.; Gu, H.; Zhang, W. Synthesis of ultrafine and highly dispersed metal nanoparticles confined in a thioether-containing covalent organic framework and their catalytic applications. *J. Am. Chem. Soc.* **2017**, *139*, 17082-17088.

(13) Liu, J.; Zhan, H.; Wang, N.; Song, Y.; Wang, C.; Wang, X.; Ma, L.; Chen, L. Palladium nanoparticles on covalent organic framework supports as catalysts for Suzuki–Miyaura cross-coupling reactions. *ACS Applied Nano Materials* **2021**, *4*, 6239-6249.

(14) Romero‐ Muñiz, I.; Mavrandonakis, A.; Albacete, P.; Vega, A.; Briois, V.; Zamora, F.; Platero‐ Prats, A. E. Unveiling the Local Structure of Palladium Loaded into Imine‐ Linked Layered Covalent Organic Frameworks for Cross‐ Coupling Catalysis. *Angewandte Chemie International Edition* **2020**, *59*, 13013-13020.

(15) Sun, Q.; Wu, C.; Pan, Q.; Zhang, B.; Liu, Y.; Lu, X.; Sun, J.; Sun, L.; Zhao, Y. Three‐ Dimensional Covalent‐ Organic Frameworks Loaded with Highly Dispersed Ultrafine Palladium Nanoparticles as Efficient Heterogeneous Catalyst. *ChemNanoMat* **2021**, *7*, 95-99.

(16) Liu, Y.; Wu, C.; Sun, Q.; Hu, F.; Pan, Q.; Sun, J.; Jin, Y.; Li, Z.; Zhang, W.; Zhao, Y. Spirobifluorene-based three-dimensional covalent organic frameworks with rigid topological channels as efficient heterogeneous catalyst. *CCS Chemistry* **2021**, *3*, 2418-2427.

(17) Tao, R.; Shen, X.; Hu, Y.; Kang, K.; Zheng, Y.; Luo, S.; Yang, S.; Li, W.; Lu, S.; Jin, Y. Phosphine‐ Based Covalent Organic Framework for the Controlled Synthesis of Broad‐ Scope Ultrafine Nanoparticles. *Small* **2020**, *16*, 1906005.

(18) Li, Y.; Wang, C.; Ma, S.; Zhang, H.; Ou, J.; Wei, Y.; Ye, M. Fabrication of hydrazone-linked covalent organic frameworks using alkyl amine as building block for high adsorption capacity of metal ions. *ACS applied materials & interfaces* **2019**, *11*, 11706-11714.

(19) Li, W.; Zhuang, Y.; Wang, J.; Yang, T.; Yu, Y.; Chen, M.; Wang, J. A three-dimensional porous organic framework for highly selective capture of mercury and copper ions. *ACS Applied Polymer Materials* **2019**, *1*, 2797-2806.

(20) Cai, Y.; Jiang, Y.; Feng, L.; Hua, Y.; Liu, H.; Fan, C.; Yin, M.; Li, S.; Lv, X.; Wang, H. Q-graphene-scaffolded covalent organic frameworks as fluorescent probes and sorbents for the fluorimetry and removal of copper ions. *Anal. Chim. Acta* **2019**, *1057*, 88-97.

(21) Jiang, Y.; Liu, C.; Huang, A. EDTA-functionalized covalent organic framework for the removal of heavy-metal ions. *ACS applied materials & interfaces* **2019**, *11*, 32186-32191.

(22) Zhou, Z.; Zhong, W.; Cui, K.; Zhuang, Z.; Li, L.; Li, L.; Bi, J.; Yu, Y. A covalent organic framework bearing thioether pendant arms for selective detection and recovery of Au from ultra-low concentration aqueous solution. *Chemical Communications* **2018**, *54*, 9977-9980.

(23) Zhao, F.; Bai, Y.; Zhou, X.; He, L.; Tao, Y.; Chen, J.; Zhang, M.; Guo, Q.; Ma, Z.; Chen, L. An Aryl‐ ether‐ linked Covalent Organic Framework Modified with Thioamide Groups for Selective Extraction of Palladium from Strong Acid Solutions. *Chemistry–A European Journal* **2023**, e202302445.

(24) Lu, Y.; Liang, Y.; Zhao, Y.; Xia, M.; Liu, X.; Shen, T.; Feng, L.; Yuan, N.; Chen, Q. Fluorescent test paper via the in situ growth of COFs for rapid and convenient detection of Pd (II) ions. *ACS applied materials & interfaces* **2021**, *13*, 1644-1650.

(25) Zhao, L.; Ma, X.; Xiong, J.; Zhou, Q.; Chen, W.; Yang, Z.; Jiang, F.; Wang, S.; Yang, X.; Bai, H. Guanidinium-functionalized ionic covalent organic framework for selective and efficient recovery of palladium (II) from metallurgical wastewater. *Journal of Environmental Chemical Engineering* **2023**, *11*, 110549.

(26) Sharififard, H.; Soleimani, M.; Ashtiani, F. Z. Evaluation of activated carbon and bio-polymer modified activated carbon performance for palladium and platinum removal. *Journal of the Taiwan institute of chemical engineers* **2012**, *43*, 696-703.

(27) Iqbal, A.; Jan, M. R.; Shah, J.; Rashid, B. Dispersive solid phase extraction of precious metal ions from electronic wastes using magnetic multiwalled carbon nanotubes composite. *Minerals Eng* **2020**, *154*, 106414.

(28) Liu, L.; Liu, S.; Zhang, Q.; Li, C.; Bao, C.; Liu, X.; Xiao, P. Adsorption of Au (III), Pd (II), and Pt (IV) from aqueous solution onto graphene oxide. *Journal of Chemical & Engineering Data* **2013**, *58*, 209-216.

(29) Lin, S.; Zhao, Y.; Bediako, J. K.; Cho, C.; Sarkar, A. K.; Lim, C.; Yun, Y. Structure-controlled recovery of palladium (II) from acidic aqueous solution using metal-organic frameworks of MOF-802, UiO-66 and MOF-808. *Chem. Eng. J.* **2019**, *362*, 280-286.

(30) Hu, C.; Xu, W.; Mo, X.; Li, H.; Zhou, S.; Zhang, P.; Tang, K. Efficient adsorption toward precious metal from aqueous solution by zeolitic imidazolate framework-8. *Adsorption* **2018**, *24*, 733-744.

(31) Lin, S.; Bediako, J. K.; Cho, C.; Song, M.; Zhao, Y.; Kim, J.; Choi, J.; Yun, Y. Selective adsorption of Pd (II) over interfering metal ions (Co (II), Ni (II), Pt (IV)) from acidic aqueous phase by metal-organic frameworks. *Chem. Eng. J.* **2018**, *345*, 337-344.

(32) Lim, C.; Lin, S.; Yun, Y. Highly efficient and acid-resistant metal-organic frameworks of MIL-101 (Cr)-NH2 for Pd (II) and Pt (IV) recovery from acidic solutions: Adsorption experiments, spectroscopic analyses, and theoretical computations. *J. Hazard. Mater.* **2020**, *387*, 121689.

(33) Tang, J.; Zhao, J.; Wang, S.; Zhang, L.; Zhao, M.; Huang, Z.; Hu, Y. Pre-modification strategy to prepare a novel Zr-based MOF for selective adsorption of Palladium (II) from solution. *Chem. Eng. J.* **2021**, *407*, 127223.

(34) Bai, F.; Ye, G.; Chen, G.; Wei, J.; Wang, J.; Chen, J. Highly selective recovery of palladium by a new silica-based adsorbent functionalized with macrocyclic ligand. *Separation and purification technology* **2013**, *106*, 38-46.

(35) Saman, N.; Kamal, N. A. A.; Lye, J. W. P.; Mat, H. Synthesis and characterization of CTAB-silica nanocapsules and its adsorption behavior towards Pd (II) ions in aqueous solution. *Advanced Powder Technology* **2020**, *31*, 3205-3214.

(36) Zhao, J.; Wang, C.; Wang, S.; Zhang, L.; Zhang, B. Augmenting the adsorption parameters of palladium onto pyromellitic acid-functionalized nanosilicas from aqueous solution. *Colloids Surf. Physicochem. Eng. Aspects* **2019**, *578*, 123581.

(37) Wei, W.; Qiu, Y.; Zhao, Y.; Zhang, K.; Ji, Y.; Gao, H.; Bediako, J. K.; Yun, Y. Development of melamine-impregnated alginate capsule for selective recovery of Pd (II) from a binary metal solution. *J. Clean. Prod.* **2021**, *288*, 125648.

(38) Yao, C.; Chen, S.; Wang, L.; Deng, H.; Tong, S. Low cost and rapid fabrication of copper sulfides nanoparticles for selective and efficient capture of noble metal ions. *Chem. Eng. J.* **2019**, *373*, 1168-1178.

(39) Song, K. S.; Ashirov, T.; Talapaneni, S. N.; Clark, A. H.; Yakimov, A. V.; Nachtegaal, M.; Copéret, C.; Coskun, A. Porous polyisothiocyanurates for selective palladium recovery and heterogeneous catalysis. *Chem* **2022**, *8*, 2043-2059.
